# Preoperative immunological plasma markers TRAIL, CSF1 and TIE2 predict survival after resection for biliary tract cancer

**DOI:** 10.3389/fonc.2023.1169537

**Published:** 2023-06-19

**Authors:** Hannes Jansson, Martin Cornillet, Dan Sun, Iva Filipovic, Christian Sturesson, Colm J. O’Rourke, Jesper B. Andersen, Niklas K. Björkström, Ernesto Sparrelid

**Affiliations:** ^1^ Division of Surgery and Oncology, Department of Clinical Science, Intervention and Technology, Karolinska Institutet, Karolinska University Hospital, Stockholm, Sweden; ^2^ Center for Infectious Medicine, Department of Medicine Huddinge, Karolinska Institutet, Karolinska University Hospital, Stockholm, Sweden; ^3^ Biotech Research and Innovation Centre (BRIC), Department of Health and Medical Sciences, University of Copenhagen, Copenhagen, Denmark

**Keywords:** cholangiocarcinoma (CCA), gallbladder cancer (GBC), prognostic biomarkers, tumor associated macrophage (TAM), biliary tract cancer (BTC)

## Abstract

**Introduction:**

Systemic inflammatory markers have been validated as prognostic factors for patients with biliary tract cancer (BTC). The aim of this study was to evaluate specific immunologic prognostic markers and immune responses by analyzing preoperative plasma samples from a large prospectively collected biobank.

**Methods:**

Expression of 92 proteins representing adaptive and innate immune responses was investigated in plasma from 102 patients undergoing resection for BTC 2009-2017 (perihilar cholangiocarcinoma n=46, intrahepatic cholangiocarcinoma n=27, gallbladder cancer n=29), by means of a high-throughput multiplexed immunoassay. Association with overall survival was analyzed by Cox regression, with internal validation and calibration. Tumor tissue bulk and single-cell gene expression of identified markers and receptors/ligands was analyzed in external cohorts.

**Results:**

Three preoperative plasma markers were independently associated with survival: TRAIL, TIE2 and CSF1, with hazard ratios (95% confidence intervals) 0.30 (0.16-0.56), 2.78 (1.20-6.48) and 4.02 (1.40-11.59) respectively. The discrimination of a preoperative prognostic model with the three plasma markers was assessed with concordance-index 0.70, while the concordance-index of a postoperative model with histopathological staging was 0.66. Accounting for subgroup differences, prognostic factors were assessed for each type of BTC. TRAIL and CSF1 were prognostic factors in intrahepatic cholangiocarcinoma. In independent cohorts, TRAIL-receptor expression was higher in tumor tissue and seen in malignant cells, with TRAIL and CSF1 expressed by intra- and peritumoral immune cells. Intratumoral TRAIL-activity was decreased compared to peritumoral immune cells, while CSF1-activity was increased. The highest CSF1 activity was seen in intratumoral macrophages, while the highest TRAIL-activity was seen in peritumoral T-cells.

**Discussion:**

In conclusion, three preoperative immunological plasma markers were prognostic for survival after surgery for BTC, providing good discrimination, even compared to postoperative pathology. TRAIL and CSF1, prognostic factors in intrahepatic cholangiocarcinoma, showed marked differences in expression and activity between intra- and peritumoral immune cells.

## 1 Introduction

Patients with biliary tract cancer (cholangiocarcinoma and gallbladder cancer) have a high risk of tumor recurrence after curative intent surgery, with poor long-term survival outcomes. A majority of patients are diagnosed with cancer recurrence within five years after surgery for cholangiocarcinoma or oncological resection for gallbladder cancer (1–4), and a median overall survival of approximately two to four years has been reported in reviews, meta-analyses and multicenter cohorts (2, 5–7). Established prognostic factors such as histopathological tumor extension, tumor grade and lymph node metastasis (2, 8, 9) are only available after tumor resection, impeding a preoperative risk stratification. Prognostic value of a systemic inflammatory response (as assessed by markers such as C-reactive protein [CRP], albumin or white cell counts) for overall survival has been indicated in several types of malignancies (e.g. colorectal cancer, pancreatic cancer, breast cancer and prostate cancer) (10), including biliary tract cancer (11). Previously, general inflammatory markers in plasma (CRP, albumin) were validated as independent negative prognostic factors for overall survival for patients with resectable biliary tract cancer (BTC) (12). The aim of this study was to identify new candidate preoperative prognostic markers and to further characterize the immune response in BTC.

## 2 Materials and methods

### 2.1 Study design

Patients undergoing primary resection for perihilar cholangiocarcinoma (pCCA), intrahepatic cholangiocarcinoma (iCCA) or gallbladder cancer (GBC) at Karolinska University Hospital, a tertiary referral center (Stockholm, Sweden), in the period January 2009 to January 2017 were assessed for inclusion in the development and internal validation cohort of this study. Patients undergoing resection for suspected BTC with benign tumors on postoperative histopathology, as well as patients with confirmed BTC found unresectable at surgical exploration, were also included as controls. The study was approved by the Regional Ethical Review Board of Stockholm and conducted in accordance with Good Clinical Practice and the Declaration of Helsinki. All patients included in the biobank provided written informed consent. The study was reported in accordance with the REMARK guidelines for prognostic studies (13), with the REMARK checklist presented in **Supplementary Table 1**. Analysis of tumor tissue expression of candidate prognostic markers and corresponding receptors/ligands was performed with gene expression data from independent and public cohorts of BTC patients, including patients from different geographic regions.

### 2.2 Sample size calculation

With two-sided p<0.05 and a power of 80%, a minimal sample size of n=88 was estimated as necessary to identify a prognostic marker with a hazard ratio of 2.0, assuming a median follow-up of 4 years, a yearly censoring ratio of 10 percent and a median overall survival of 24 months for unexposed patients (12, 14).

### 2.3 Patient inclusion

**Figure 1:**
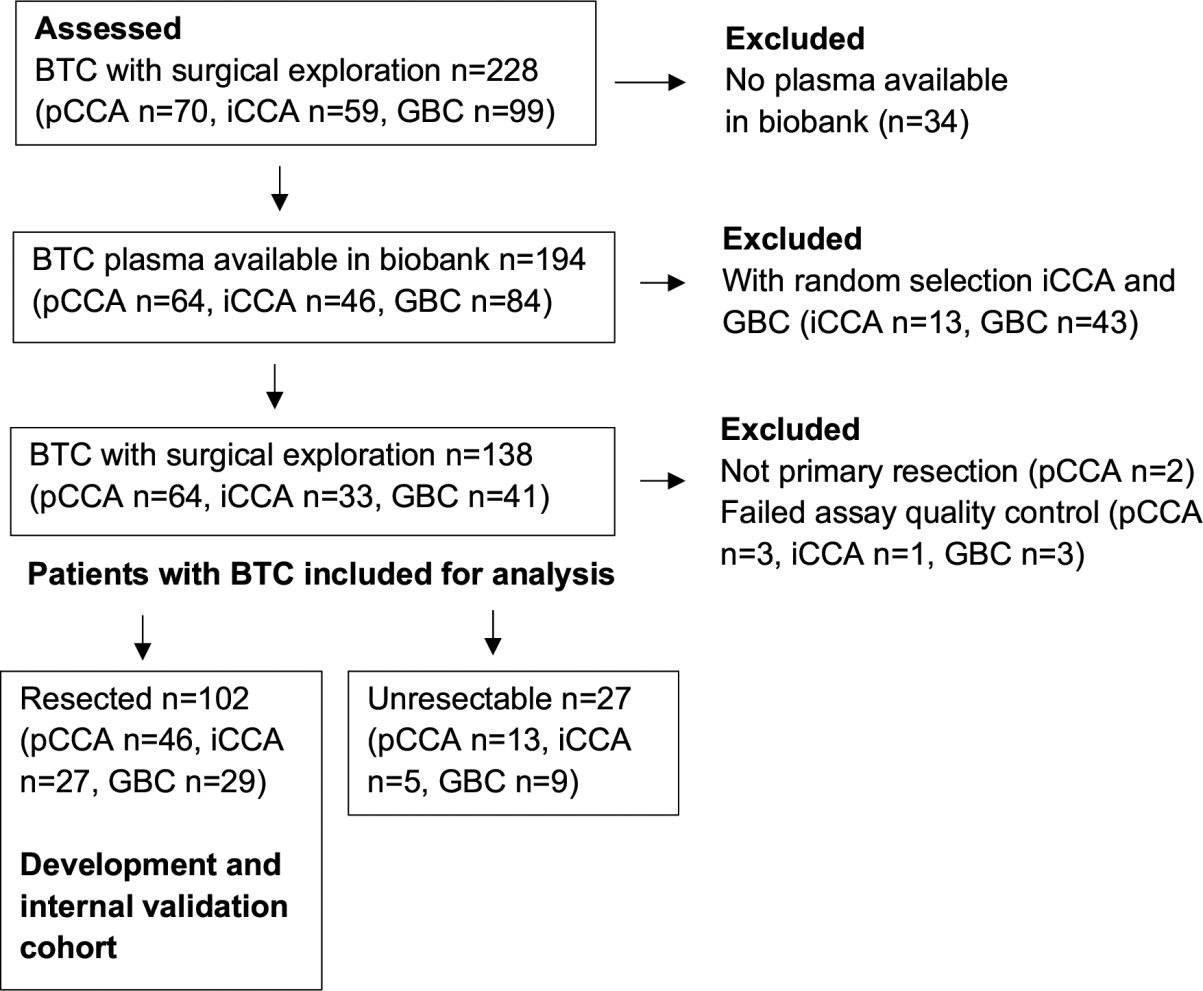
Study flow chart for the inclusion of patients with BTC. BTC, biliary tract cancer; GBC, galibladder cancer; iCCA, intrahepatic cholangiocarcinoma; pCCA, perihilar cholangiocarcinoma.

One-hundred and seven patients operated with primary resection for BTC were selected for inclusion in the development and internal validation cohort: all resected pCCA patients with plasma samples available in biobank (resected confirmed pCCA n=47), and random samples from all patients operated for iCCA (resected confirmed iCCA n=28) and GBC (resected confirmed GBC n=32). Furthermore, 29 patients with confirmed BTC found unresectable on exploration and 32 patients resected on suspicion of BTC with a benign lesion on final postoperative histopathology were included. Two patients not operated with primary resection (one case of re-resection and one patient undergoing transplantation) were excluded from analysis, as well as seven patients where samples did not pass internal quality control for the proximity extension assay (resectable BTC n=5, unresectable BTC n=2). Finally, 102 resected patients with confirmed BTC, 27 patients with confirmed BTC found unresectable and 32 patients resected with a benign lesion on final postoperative histopathology were included for analysis. The study flow chart for BTC patients is presented in **Figure 1**.

### 2.4 Sample preparation and multiplex immunoassay analysis

EDTA plasma samples were collected preoperatively at the day of surgery, centrifuged, aliquoted, frozen and stored at -80° Celsius. For Proximity Extension Assay-analysis (PEA), plasma samples were thawed on ice, and 20 microliters transferred to 96 well plates. PEA employs paired oligonucleotide coupled antibodies for detection of each analyte, with relative quantification of protein expression by polymerase chain reaction (PCR) (15). The full panel of analytes for the PEA (Immuno-Oncology I) is presented in **Supplementary Table 2**. Internal quality control of the immunoassay, extension and detection steps in each sample was performed with assay-specific protein-, antibody- and double stranded oligonucleotide controls respectively, while interplate control was performed with a set of 92 oligonucleotide duplexes. Relative quantification for each analyte by PEA was expressed as Normalized Protein Expression units (NPX) in Log2 scale, after normalization of PCR quantification cycle values for intra- and interassay variation using the detection and interplate controls. PEA analysis was performed at an institutional core facility (SciLifeLab, Clinical Biomarker Facility, Uppsala University, Uppsala, Sweden) blinded to all outcome data. The PEA has been validated for preserved analytical precision with hyperlipidemia and hyperbilirubinemia corresponding to 8-10 times upper reference values (16). No patient in the development and internal validation cohort underwent surgery with a bilirubin >190 micromoles/litre. The Immuno-Oncology I-panel has also been validated for interference of hemolysate in plasma, allowing up to 5-10% hemolysis of a sample for reliable detection of 84 of 92 proteins, while eight proteins in the panel were identified as more sensitive for interference by hemolysate (Adenosine deaminase, Arginase-1, Caspase-8, C-X-C-motif chemokine 11, Galectin-9, Granzyme-B, Granzyme-H and Interleukin-18) (16).

### 2.5 Outcome variables and clinicopathological data

The primary outcome was overall survival calculated from the date of surgery. Clinical data were retrospectively collected from quality registries and the electronic health record. Last follow-up was 11 Aug 2019. Demographic and clinicopathological variables collected were: age, sex, preoperative physical status classification according to the American Society of Anesthesiologists, diagnosis of primary sclerosing cholangitis, cirrhosis or diabetes, tumor extension stage, lymph node metastasis (N1), lymphovascular- and perineural invasion, microscopically tumor-positive resection margin (R1) and tumor differentiation (grade). Histopathological staging was reported according to the 7^th^ edition of the AJCC/TNM guidelines and tumor grade according to the College of American Pathologists (17, 18).

### 2.6 Gene expression analyses

The following gene expression datasets were analyzed: GSE107943 (19), GSE138709 (20), GSE89749 (21), GSE26566 (22) (Gene Expression Omnibus), EGAD00001001693 (23) (European Genome-Phenome Archive, study ID: EGAS00001000950), E-MTAB-6389 (24) (ArrayExpress), OEP001105 (25) (Biosino), phs001404.v1.p1 (26) (dbGaP) and HRA000863 (27) (Genome Sequence Archive). Differential expression was analyzed using limma 3.50.0 (28) for microarray data and DESeq2 1.34.0 (29) for sequencing data. For single-cell RNA-sequencing data from HRA000863, raw BAM files were converted back to FASTQ format using the CellRanger 6.1.2 (30) *bamtofastq* command and read counts per gene per cell were obtained by CellRanger *count (* 30 *)*. For processed expression data from GSE138709 and HRA000863, analysis was performed in R 4.1.1 with the Seurat 4.0.4 package (31). Data were normalized and scaled after filtering out cells with gene counts below 500 or greater than 3 000, as well as cells with a percentage of mitochondrial genes above 5. Data from different samples were then integrated by Harmony (32). For HRA000863 (27) and GES138709 (20) datasets, a total of 239 760 and 28 261 cells were clustered by principal component analysis and visualized with uniform manifold approximation and projection (UMAP), respectively. Clusters were annotated by mapping to references for immune cells according to CITE-seq data (31), annotation of malignant cells according to copy number variation (CNV) scores (with a cut off score of 3 for malignancy) calculated using InferCNV 1.8.1 (33), and by using cell markers for hepatocytes (not present in HRA000863), cholangiocytes, fibroblasts and endothelial cells (20). Differential expression of biomarkers between different cell types or between cells from tumor and periphery were tested using FindMarkers() with logfc.threshold and min.pct set to 0. Modelling of cytokine activities from single-cell transcriptome profiles were performed using the Cytokine Signaling Analyzer (CytoSig) v0.1 (34). Specifically, counts per gene were first converted to transcripts per million (TPM) and log_2_-transformed, and expression values across all cells were mean centralized. Permutation tests were used to compare activity Z-scores obtained from Cytosig between tumor and periphery samples. That is, after obtaining the mean of Z-scores of a particular cell type for either tumor or periphery samples; that mean was compared to the mean of same number of cells randomly chosen (with replacement) from that cell type regardless of sample location. This process was repeated 10 000 times, and an empirical p-value was calculated as [10 000 - N_Mean_real >Mean_permutation_]/10 000.

### 2.7 Statistical analysis

Statistical analyses were performed in R (R 3.5.3 and 4.1.1, R Foundation for Statistical Computing; RStudio 1.1.463, 1.4.1717 and 2021.09.0, RStudio Inc, Boston, USA), SPSS Statistics v25 and v28 (IBM, New York, USA) and Olink Insights Stat Analysis (Olink Proteomics, Uppsala, Sweden). Inclusion of iCCA and GBC patients was performed with random sampling from all consecutively operated iCCA and GBC patients respectively in SPSS. Imputation of missing data was used for independent variables included in regression analysis. For proteomics data, values below the limit of detection were imputed as left-censored data missing not at random by a quantile regression method (35). For other variables, multivariate imputation was performed (36). Demographic and clinicopathological characteristics at baseline were reported with unimputed data. Correlations among variables were assessed with Spearman’s rank correlation, and visualized with heatmaps after hierarchical clustering according to the degree of correlation (37). For Cox regression analysis, the proportionality of hazards assumption was tested with scaled Schoenfeld residuals (38). To account for multiple comparisons in evaluation of univariable prognostic value, the Bonferroni-Holm corrected p-values were calculated and variables with an adjusted univariable p-value <0.20 were included in multivariable models. For variable selection in Cox regression modelling, backward elimination was applied with stopping criterion unadjusted p=0.157, equivalent to the Akaike information criterion (39). Differential protein expression between patient subgroups was analyzed by independent t-test, with corrected p-values according to the Benjamini-Hochberg method and illustrated with volcano plots. Additionally, non-parametric analysis was performed by Mann-Whitney U test.

The discriminatory ability of multivariable prognostic models was assessed with concordance indices (c-index) where a c-index of 0.50 would indicate no predictive ability and a c-index of 1.00 would indicate perfect predictive ability (40). The calibration of predictions for specified time points was assessed with calibration curves (40). To account for overfitting, internal validation of multivariable models by bootstrap resampling was performed (resamples n=600) (40). For survival analysis with Kaplan-Meier curves and Cox regression, SPSS and in R the survival and rms packages were used (38, 40). Survival curves were compared using the log-rank test. For survival analyses with gene expression data, patients were stratified into groups according to marker expression above/below the median. Significance tests were all two-sided and p-values <0.05 were considered statistically significant.

## 3 Results

Baseline characteristics and clinicopathological variables for the 102 included patients resected for BTC and 27 patients with unresectable BTC are presented in **Table 1**. There were 75 deaths during a median follow-up of 67 months (95% CI 55-79 months) among the 102 resected BTC patients, while all 27 patients found unresectable at exploration were followed to death. No patients were censored before 24 months after surgery. Median overall survival was 20 months (95% CI 16-24 months) for all BTC patients, 23 months for resected patients (95% CI 17-29 months) and 7 months for unresectable patients (95% CI 0-14 months).

**Table 1:** Demographic and clinicopathological characteristics of BTC patients.

Variable	BTC resected n=102	Missing data BTC resected	BTC unresectable n=27	Missing data BTC unresectable	p-value
Age Y, md (IQR)	66 (54-71)	–	65 (60-70)	–	0.81^$^
Sex F, n (%)	52 (51)	–	14 (52)	–	0.94
BMI, md (IQR)	25 (23-29)	–	24 (23-30)	–	0.94^$^
Diabetes, n (%)	13 (13)	–	5 (19)	–	0.53^&^
Cirrhosis, n (%)	5 (5)	–	2 (7)	–	0.64^&^
ASA≥3, n (%)	30 (29)	–	8 (30)	–	0.98
GPS≥1, n (%)	55 (54)	17	20 (74)	6	0.007*
PSC, n (%)	12 (12)	–	1 (4)	–	0.30^&^
PVE, n (%)	18 (18)	–	3 (11)	–	0.56^&^
BTC subgroup:					0.68
pCCA, n (%)	46 (45)	–	13 (48)	–	
iCCA, n (%)	27 (27)	–	5 (19)	–	
GBC, n (%)	29 (28)	–	9 (33)	–	
Major resection, n (%)^#^	73 (72)	–			
CD≥3, n (%)^#^	47 (46)	–			
Postoperative mortality, n (%)^#^	10 (10)	–			
T≥3, n (%)^#^	45 (44)	1			
N1, n (%)^#^	49 (48)	12			
Pn1, n (%)^#^	73 (72)	7			
LV1, n (%)^#^	77 (75)	7			
R1, n (%)^#^	65 (64)	2			
Grade≥2, n (%)^#^	79 (77)	9			

ASA, American Society of Anesthesiologists; BMI, body mass index; BTC, biliary tract cancer; CD, Clavien-Dindo complication grade; F, female; GBC, gallbladder cancer; GPS, Glasgow prognostic score; iCCA, intrahepatic cholangiocarcinoma; IQR, interquartile range; LV1, lymphovascular invasion; md, median; N1, lymph node metastasis; pCCA, perihilar cholangiocarcinoma; Pn1, perineural invasion; Postoperative mortality, in-hospital postoperative mortality (not limited to 90 days). PSC, primary sclerosing cholangitis; PVE, portal vein embolization; R1, microscopically tumor positive resection margin; T, tumor extension; Y, years.

#: Reported for resected patients; $: Mann-Whitney U; &: Fisher Exact test; * p<0.05.

### 3.1 Analysis of plasma protein expression

Of the 92 proteins analyzed by PEA, 14 proteins were not detected in >75% of samples. A list of the 78 proteins included for further analysis is presented in **Supplementary Table 3**. No proteins were differentially expressed between resected and unresectable BTC patients (**Supplementary Table 4**).

**Figure 2:**
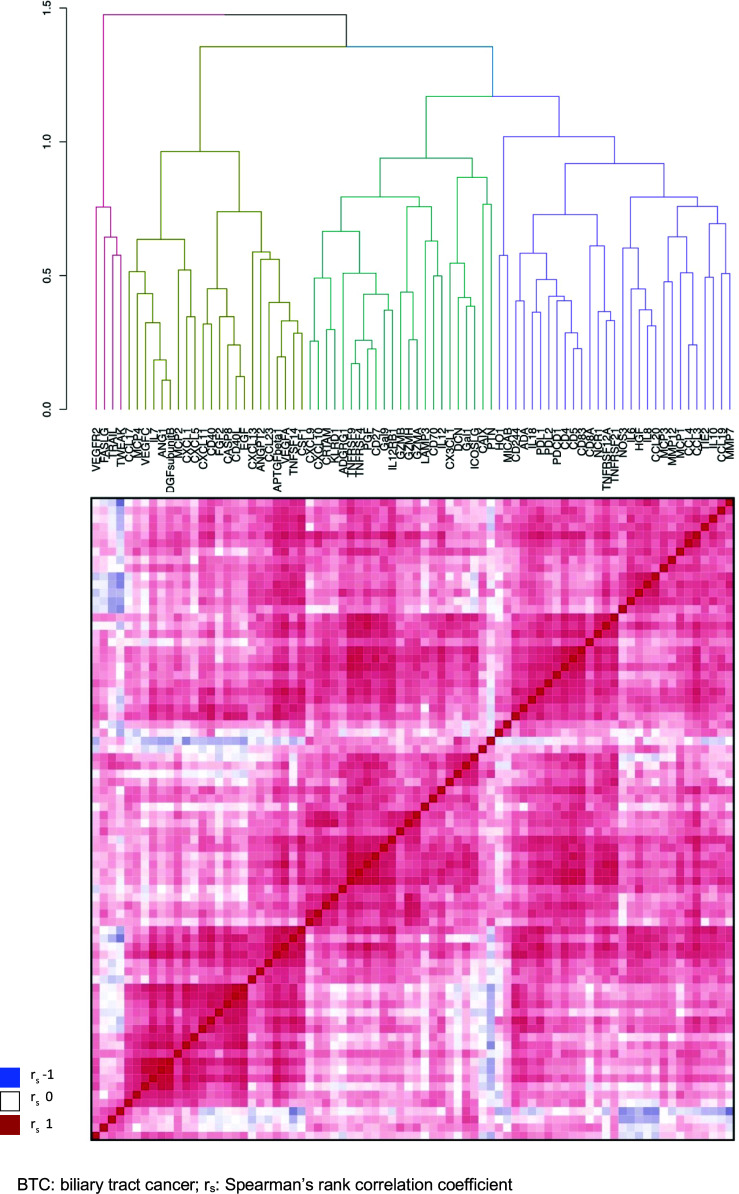
Correlation matrix for the expression of 78 proteins in plasma of patients with BTC, hierarchically clustered. BTC, biliary tract cancer; rs: Spearman's rank correlation coefficient.

To illustrate correlation of expression and identify non-redundant candidate markers, all proteins analyzed in BTC patients were grouped by hierarchical clustering, according to the degree of correlation. Two main clusters were formed (**Figure 2**; **Supplementary Table 5**), with the larger cluster subdivided into three subgroups. The smaller main cluster (cluster 1) contained three proteins related to the external induction of apoptosis, together with VEGFR2. The larger main cluster (cluster 2) contained proteins including effector molecules, chemokines, mitogens and other regulators of immune cell proliferation and differentiation.

The correlation of the plasma proteins with other clinicopathological variables and established prognostic factors was also evaluated and is illustrated in **Supplementary Figure 1**. Demographic and clinicopathological variables (age, sex, tumor stage, lymph node metastasis, perineural invasion, lympho-vascular invasion, tumor grade) were not internally strongly correlated, with the strongest correlation found between tumor stage and tumor grade (r = 0.37) and between lympho-vascular and perineural invasion (r = 0.27). The strongest correlation between demographic/clinicopathological variables and plasma proteins analyzed by PEA was seen between age and Pleiotrophin (PTN, r = 0.59).

### 3.2 Uni- and multivariable survival analysis

Association of the 78 proteins with overall survival after resection surgery was investigated by univariable Cox regression analysis (**Supplementary Table 6**). Six proteins were found to be associated to overall survival with univariable adjusted p-value <0.20 (unadjusted p-value <0.005) and are presented in **Table 2** and with Kaplan-Meier curves in **Supplementary Figure 2**.

**Table 2:** Uni- and multivariable Cox regression analyses (resected BTC n=102).

Variable	Univariable HR (95% CI)	p-value unadjusted (adjusted)	Multivariable HR (95% CI)	p-value all	Multivariable HR (95% CI) selected	p-value selected
TRAIL	0.35 (0.18-0.67)	<0.001* (0.096)^§^	0.29 (0.14-0.59)	<0.001*	0.30 (0.16-0.56)	<0.001*
TNFSF14	1.84 (1.33-2.54)	<0.001* (0.015)*	1.13 (0.71-1.78)	0.61		
CSF1	6.52 (2.42-17.54)	<0.001* (0.013)*	4.04 (1.03-15.81)	0.045*	4.02 (1.40-11.59)	0.010*
IL6	1.31 (1.10-1.56)	0.003* (0.15)^§^	0.92 (0.72-1.20)	0.55		
IL8	1.34 (1.13-1.59)	<0.001* (0.056)^§^	1.00 (0.77-1.30)	0.98		
TIE2	4.33 (1.97-9.51)	<0.001* (0.016)*	2.82 (1.09-7.32)	0.033*	2.78 (1.20-6.48)	0.018*

BTC, biliary tract cancer; CSF1, colony-stimulating factor 1; IL6, interleukin 6; IL8, interleukin 8; CI, confidence interval; HR, hazard ratio; TIE2, tyrosine kinase with immunoglobulin-like and EGF-like domains 2; TRAIL, TNF-related apoptosis-inducing ligand.

* p<0.05; ^§^ p<0.20.

The protein with a positive association to survival was located in cluster 1 (TRAIL/TNFSF10, death receptor ligand, one of the effector mechanisms of macrophages and NK-cells), and five proteins with a negative association were located in two subgroups of cluster 2 (TNFSF14, an effector and regulator of T-cell activity; CSF1/M-CSF, a regulator of monocyte proliferation, differentiation and function; IL6, inducer of acute phase response and regulator of lymphocyte and monocyte differentiation; IL8, chemotactic for neutrophils, basophils and T-cells; and TIE2/TEK, angiopoietin receptor and a regulator of angiogenesis). The six proteins associated with overall survival were included in multivariable analysis (events per variable 75/6 = 12.5) with three proteins selected by backward elimination (**Table 2**), representing separate clusters/subgroups in the hierarchical clustering analysis (TRAIL: cluster 1, CSF and TIE2: separate subgroups cluster 2).

### 3.3 Discrimination of pre- and postoperative prognostic models

The discriminatory ability of the three preoperative candidate markers TRAIL, CSF1 and TIE2 for overall survival after resection was assessed with a c-index of 0.71 for the three markers combined. C-indices for the separate markers were 0.61, 0.65 and 0.63 for TRAIL, CSF1 and TIE2 respectively. The prognostic ability of postoperative pathology (T-stage, N-status, perineural invasion, lympho-vascular invasion, tumor grade, resection margin) was assessed with a c-index of 0.70. Adding the three preoperative candidate markers to a combined model with postoperative pathology increased the c-index to 0.74. Internal validation of the prognostic value was performed with bootstrap correction, where the corrected c-index for the three preoperative candidate markers was 0.70, while the corrected c-index for postoperative pathology was 0.66. The corrected c-index for a model with the three preoperative candidate markers added to postoperative pathology was 0.72.

A validated preoperative prognostic factor, Glasgow prognostic score (GPS, calculated from albumin and CRP concentrations: CRP>10 mg/L or albumin<35 g/L = 1 point each), was analyzed with a corrected c-index of 0.65. In the analysis of correlation of plasma protein expression and other clinicopathological/prognostic variables, there was a moderate correlation between GPS and CSF1 (r=0.49) and between GPS and TIE2 (r=0.42), where GPS and TIE2 grouped together in hierarchical clustering (**Supplementary Figure 1**).

A preoperative prognostic model including the three candidate markers together with the GPS was assessed with a c-index of 0.71, and a bootstrap corrected c-index of 0.69. A postoperative prognostic model including pathological variables together with GPS was assessed with c-index 0.75, and corrected c-index of 0.71. Multivariable models including both pre- and postoperative factors are presented in **Supplementary Table 7**.

### 3.4 Calibration of preoperative prognostic markers

The calibration of a preoperative prognostic model with the three candidate markers TRAIL, CSF1 and TIE2 was assessed for one-, three- and five-year overall survival, as illustrated with calibration plots in **Supplementary Figure 3** (bootstrap corrected preoperative models indicated by the blue lines, uncorrected models indicated by the black lines). Actual survival at one year was lower than predicted by the preoperative model (**Supplementary Figure 3A**), while at three years and five years the model underestimated survival predicted below 60% and 40% respectively (**Supplementary Figures 3B, C**).

### 3.5 Subgroup analyses and internal validation of disease specific prognostic models

The prognostic value of the three identified plasma markers within each BTC subgroup (iCCA, pCCA and GBC) was further analyzed (**Supplementary Table 8**). For the iCCA group, TRAIL and CSF1 retained prognostic value while TIE2 (p=0.52) did not. For the pCCA group, TRAIL was a significant prognostic factor while TIE2 (p=0.05) and CSF1 (p=0.17) were not. For gallbladder cancer, CSF1 and TIE2 but not TRAIL (p=0.15), remained prognostic.

The prognostic performance of three disease-specific preoperative prognostic models including GPS was evaluated with bootstrap correction to account for overfitting. The corrected c-indices for models specific for iCCA (TRAIL, CSF1, GPS), pCCA (TRAIL, TIE2, GPS) and GBC (CSF1, TIE2) were 0.78 (uncorrected 0.80), 0.65 (uncorrected 0.68) and 0.74 (uncorrected 0.75) respectively. A cholangiocarcinoma-specific (iCCA + pCCA) prognostic model with only TRAIL and GPS had a c-index of 0.69 (uncorrected 0.69).

### 3.6 Comparison of plasma protein expression in BTC and benign controls

The differential expression of plasma proteins between BTC patients and patients with benign histopathology after resection for suspected BTC was analyzed, with expression levels of 25 proteins significantly higher and with no proteins showing lower expression in patients with BTC (**Supplementary Table 9**; **Supplementary Figure 4**). CSF1 and TIE2, but not TRAIL, was higher in patients with malignancy. Excluding TRAIL from the preoperative prognostic model for patients with BTC did not improve discrimination (c-index 0.65, bootstrap corrected c-index 0.64).

The five proteins found with higher levels of expression in malignancy and with the most statistically significant difference compared to patients with benign lesions were IL6, PGF, CSF1, MMP12 and HGF, with a significant difference also on non-parametric testing (PGF, CSF1, MMP12: p<0.001; IL6, HGF: p=0.004). There was a considerable overlap in expression levels for these proteins between the benign group and the BTC group (**Supplementary Figure 5A**). CSF1, PGF and MMP12 had the highest area under the receiver operating curve values for predicting malignancy (all: AUROC=0.69), with CSF1 and PGF showing slightly better performance according to precision-recall curve analysis (**Supplementary Figure 5B**).

### 3.7 Analysis of tumor tissue-specific expression of plasma markers and receptors/ligands

**Figure 3:**
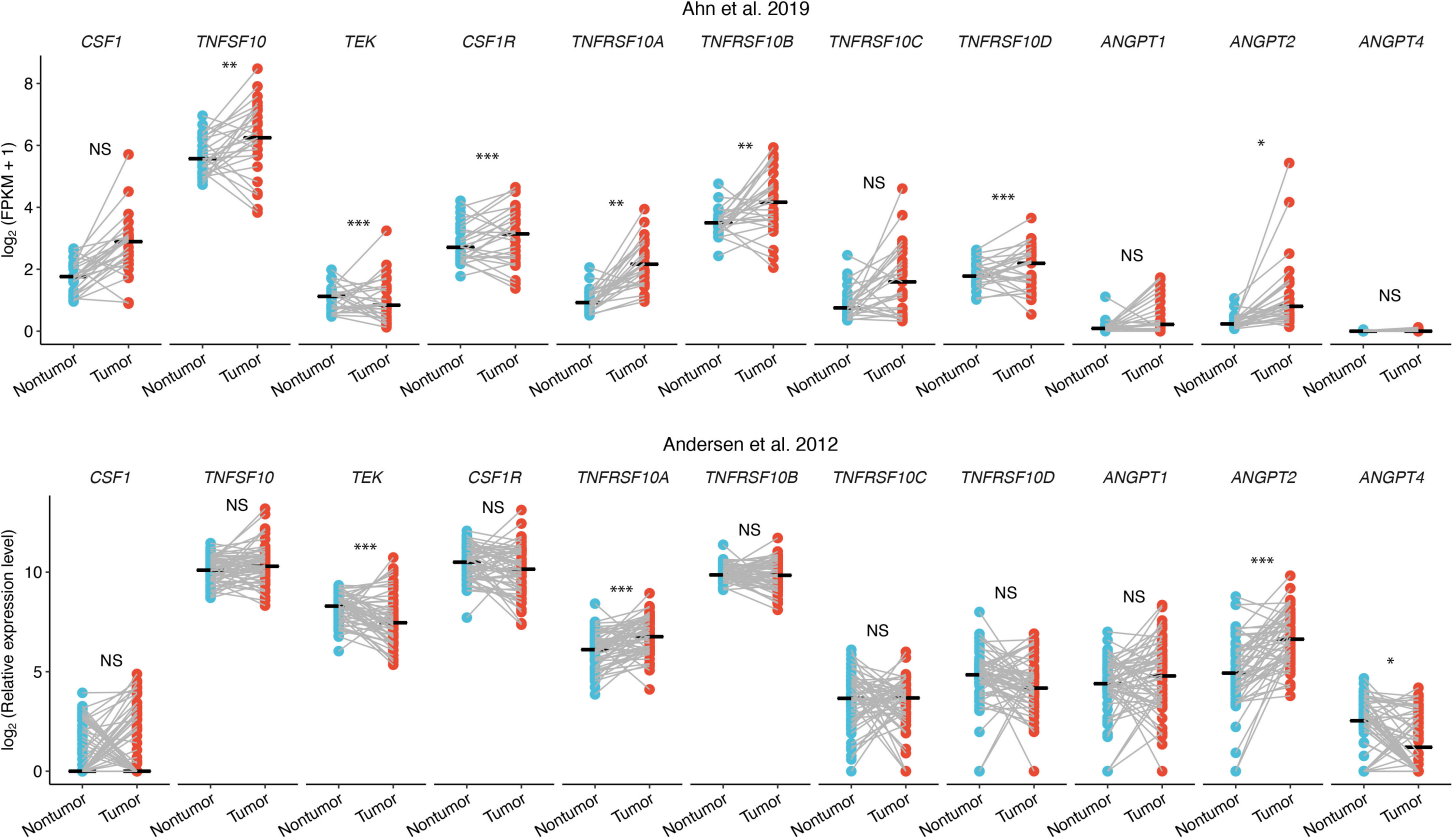
Differential gene expression of markers and ligands/receptors in CCA tissue, tumor tissue (red) and surrounding liver (blue). NS, not significant; * adjusted p<0.05; ** adjusted p<0.01; *** adjusted p<0.001.

The tumor tissue-specific expression of the three identified plasma markers and their respective receptors (*CSF1*: *CSF1-R*; *TRAIL*: *TRAIL-R1*/*TNFRSF10A*, *TRAIL-R2*/*TNFRSF10B*, *TRAIL-R3*/*TNFRSF10C* and *TRAIL-R4*/*TNFRSF10D*) or ligands (*TIE2*/*TEK*: *ANGPT1*, *ANGPT2* and *ANGPT4*) was then analyzed with gene expression data from two external surgical CCA cohorts with samples included from both tumor and normal surrounding liver: GSE107943 published by Ahn et al. (19) (Korea, sequencing, n=30, iCCA, hepatitis B/C 13%, recurrence and survival data with median follow-up 30.5 months) and GSE26566 published by Andersen et al. (22) (USA, Belgium and Australia, microarray, matched samples n=58, iCCA and pCCA) (**Figure 3**).

Seven out of the 11 genes analyzed were differentially expressed in tumor compared to surrounding liver in the GSE107943 dataset, and expression levels of three of the same seven proteins were likewise higher (*TRAIL-R1*/*TNFRSF10A*, *ANGPT2*) or lower (*TIE2*/*TEK*) in tumors in the GSE26566 dataset (**Figure 3**).

### 3.8 Cell type-specific expression of markers and receptors/ligands in tumor tissue

**Figure 4:**
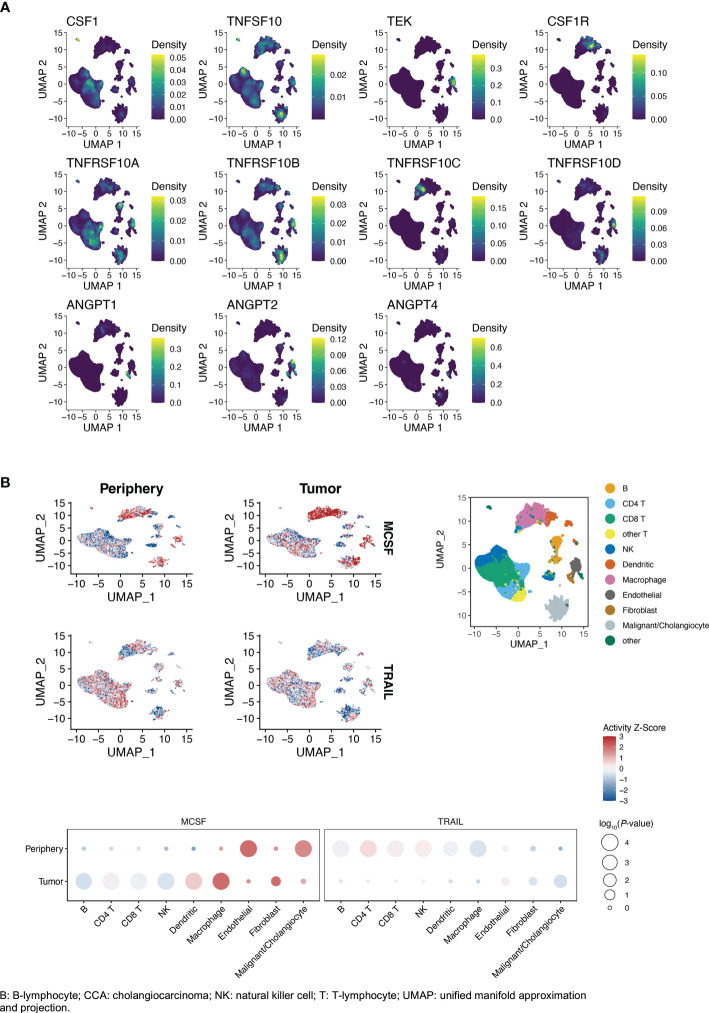
**(A, B)** Single-cell gene expression of markers and receptors/ligands in iCCA (Song et al. (27)), clusters by cell type (right top panel **B**). **(B)** Cytokine activity intratumorally vs. peritumorally. Activity Z-scores trimmed to [-3, 3] to facilitate visualization. P-values in balloon plots calculated by permutation tests (see Patients and methods).

By interrogation of single-cell gene expression data for iCCA in two datasets, published by Song et al. (27) (China, tumor samples n=14 [from patients n=14]/surrounding non-tumor liver samples n=14, hepatitis B 29%) and Zhang et al. (20) (China, tumor samples n=5 [from patients n=4]/surrounding non-tumor liver samples n=3, hepatitis B 50%), the cell type-specific expression of markers and their receptors or ligands was examined (**Figure 4**; **Supplementary Tables 10–14**).

In both datasets, expression of *TRAIL* and *TRAIL-R1*/*TNFRSF10A* was higher in malignant cells, compared to the average of other cell types in tumor and surrounding liver tissue (**Supplementary Tables 11, 12**). *TRAIL* was similarly highly expressed by monocytes, T-cells, cholangiocytes and endothelial cells (**Figure 4A**; **Supplementary Table 10**). Expression of *TRAIL-R2*/*TNFRSF10B* and *TRAIL-R4*/*TNFRSF10D* was significantly higher in endothelial cells compared to the average of other cell types (**Figure 4A**; **Supplementary Tables 10–12**). *TRAIL-R2*/*TNFRSF10B* was expressed by a large fraction of the malignant cells (Song et al: 26.3%, Zhang et al: 26.1%), and at higher average levels than the other *TRAIL* receptors (**Supplementary Tables 10–12**).

**Figure 5:**
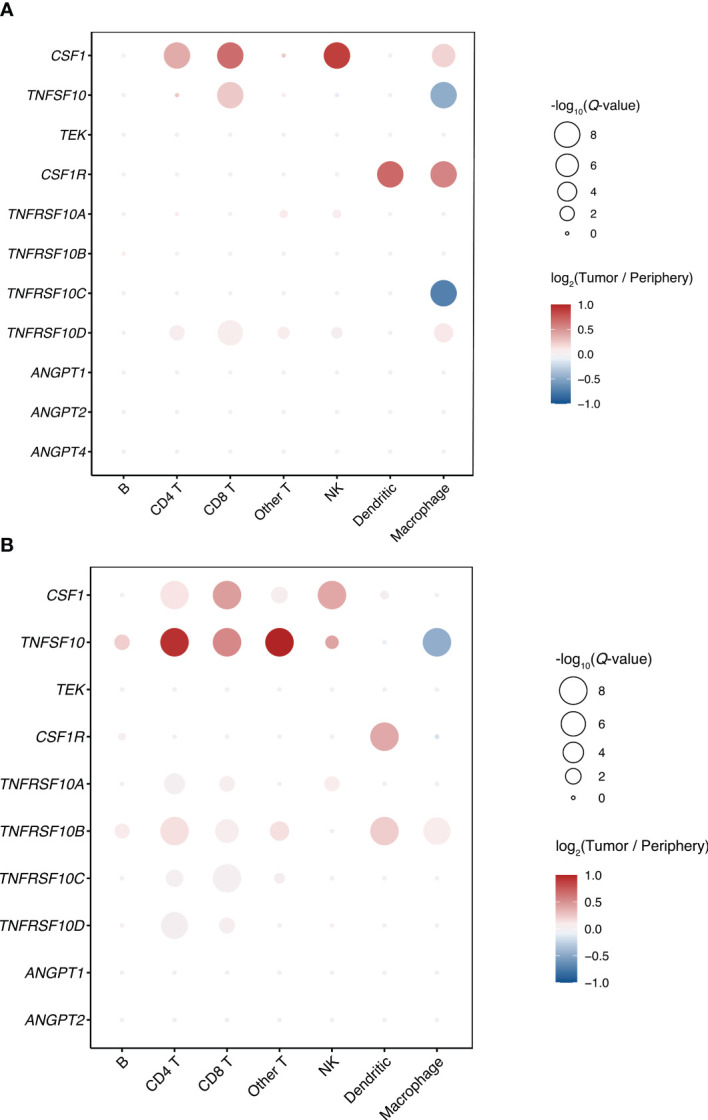
Differences in gene expression of markers and receptors/ligands between intratumoral and peritumoral immune cells in iCCA. **(A)** Song et al. (27) **(B)** Zhang et al. (20).

*TIE2*/*TEK* was mainly expressed by endothelial cells and *TIE2*/*TEK* ligands *ANGPT1* and *ANGPT2* were mainly expressed by fibroblasts (**Supplementary Tables 10–12**). *CSF1* was most highly expressed by T-cells, NK-cells, fibroblasts and endothelial cells. When comparing intratumoral immune cells to the same immune cell type in surrounding liver, *TRAIL* expression was higher in intratumoral CD8+ T-cells, but significantly lower in intratumoral macrophages compared to macrophages outside of the tumor (**Figure 5**; **Supplementary Tables 13, 14**). Expression of *CSF1* was significantly higher in intratumoral CD8+/CD4+ T-cells and NK-cells compared with T-cells and NK-cells in surrounding liver.

Comparing the cytokine activities of intra- and peritumoral immune cells in the larger Song et al. dataset, CSF1 activity was generally increased intratumorally, while TRAIL activity was generally decreased (**Figure 4B**). The highest immune cell CSF1 activity was seen in intratumoral macrophages, while the highest TRAIL activity was seen in peritumoral T-cells. The TRAIL activity in tumor cells and intratumoral cholangiocytes was higher compared to peritumoral cholangiocytes.

Similarly, in the Zhang et al. dataset, the highest immune cell CSF1 activity was seen in intratumoral macrophages, while the highest TRAIL activity in immune cells was seen in peritumoral T-cells (**Supplementary Figure 6**). In both single-cell datasets, the highest non-immune cell tumor stroma TRAIL activity was seen in endothelial cells.

### 3.9 Prognostic influence of tumor tissue expression of markers and receptors/ligands

**Figure 6:**
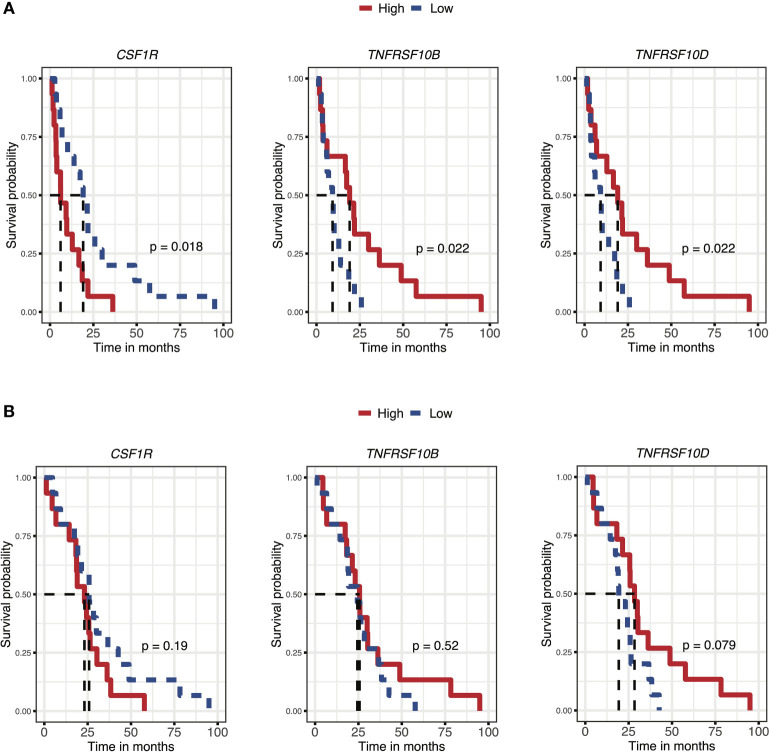
Prognostic influence of iCCA tumor tissue gene expression of markers and receptors/ligands, for disease-free survival **(A)** and overall survival **(B)** (Ahn et al. (19)).

The prognostic influence of tumor tissue expression of the identified markers and their receptors or ligands was analyzed using recurrence and survival data available for the GSE107943 dataset (19) (**Figure 6**, disease-free survival 6A, overall survival 6B; **Supplementary Figure 7**). Expression levels of three receptors (*CSF1-R* p=0.02, *TRAIL-R2*/*TNRFRSF10B* p=0.02, *TRAIL-R4*/*TNFRSF10D* p=0.02) were associated to disease-free survival, while no significant association was seen to overall survival for these genes (*CSF1-R* p=0.19, *TRAIL-R2*/*TNRFRSF10B* p=0.52, *TRAIL-R4*/*TNFRSF10D* p=0.08). Survival analyses according to expression of the remaining receptors and ligands are presented in **Supplementary Figure 7** (disease-free survival 7A, overall survival 7B). The disease-free and overall survival curves stratified according to expression of *TRAIL-R1*/*TNFRSF10A* did not reach statistical significance (disease-free survival p=0.08, overall survival 0.07). While *CSF1-R* was negatively associated to disease-free survival, expression of *TRAIL-R2*/*TNFRSF10B* and *TRAIL-R4*/*TNFRSF10D* was positively associated to disease-free survival.

For further investigation of BTC tumor tissue expression and the possible prognostic influence in a wider setting, gene expression data from six additional cohorts was interrogated. Three datasets represented diverse iCCA cohorts, from Japan (23) (Nakamura et al, RNA sequencing, n=112, hepatitis B 5%, hepatitis C 3%), France (24) (Job et al, n=72, microarray, hepatitis B 5%, hepatitis C 3%) and China (25) (Dong et al, RNA sequencing, n=224, alpha-fetoprotein [AFP] ≥21ng/ml 10%, hepatitis B 27%). Two datasets represented multinational mixed cholangiocarcinoma cohorts: Jusakul et al. (21) (Singapore, Thailand, South Korea, Romania, France, Brazil; microarray, iCCA/pCCA/distal CCA/extrahepatic CCA, n=115, fluke positive 43%, hepatitis B 8%, hepatitis C 3%) and Andersen et al. (22) (Australia, Belgium, France, Germany, Italy, USA, microarray, iCCA/pCCA, expanded cohort n=178, hepatitis C 4%). One dataset, from Nepal et al. (26), represented a multinational GBC cohort (China, Chile; RNA sequencing, n=44, hepatitis B 4%). While the indicated positive prognostic value for disease-free survival of *TRAIL-R* expression in iCCA tumor tissue in the Ahn et al. cohort was supported by overall survival data from the Job et al. cohort (*TRAIL-R1*/*TNFSFR10A* p=0.03, *TRAIL-R2*/*TNFSFR10B* p=0.16, *TRAIL-R4*/*TNFSFR10D* p=0.006), such an association was not seen in the cohorts from Nakamura et al. or Dong et al. (**Supplementary Figure 8**). In the later cohort instead, a negative association to overall survival was seen for *TRAIL-R1*/*TNFSFR10A* (p=0.005) and *TRAIL-R4*/*TNFSFR10D* (p=0.04). A negative prognostic value was seen for tumor tissue *CSF1* expression only in the cohort from Nakamura et al. (p=0.047). No other associations to survival were seen.

## 4 Discussion

Long term survival outcomes for patients undergoing resection for BTC remain poor, with a median overall survival of approximately two to four years. While multimodal therapy is under current development, prognostic factors to allow preoperative risk stratification and development of better tailored treatments remain ill-defined.

In a previous study, general inflammatory markers were validated as preoperative prognostic factors (12). The present analysis, of samples from a unique prospectively collected biobank, was therefore aimed at identifying more specific immunologic prognostic markers and to better characterize immune responses in BTC.

By means of a high-throughput multiplexed immunoassay three candidate preoperative plasma markers were identified, with a combined prognostic value for survival similar to that of established postoperative pathology. TRAIL/TNFSF10 was identified as a positive prognostic factor in both iCCA and pCCA. CSF1/M-CSF was identified as a negative prognostic factor in iCCA and GBC. TIE2/TEK was identified as a significant negative prognostic factor in GBC.

To clarify the tumor-specific expression of the identified prognostic markers and receptors or ligands, analyses were performed across four separate datasets investigating BTC tissues and surrounding liver by means of microarray, next-generation sequencing and single-cell sequencing. Over all three platforms and in demographically diverse cohorts, higher *TRAIL-R1*/*TNFSFR10A* was seen in tumor tissue/cholangiocarcinoma cells. With single-cell analysis of iCCA tissues from two separate cohorts, higher *TRAIL-R1*/*TNFRSF10A* expression was seen specifically in malignant cells. The ligand *TRAIL*/*TNFSF10* was expressed by intratumoral T-cells, B-cells, NK-cells, monocytes, malignant cells, normal cholangiocytes and endothelial cells. The expression of *TRAIL*/*TNFSF10* was higher in intratumoral CD8+ T-cells as compared to CD8+ T-cells in surrounding tissue, but decreased in intratumoral macrophages. *CSF1*/*M-CSF* was expressed by T-cells, NK-cells, fibroblasts and endothelial cells. This altogether suggests a possible important role of a T-cell-/NK-cell/monocyte-mediated TRAIL-R1/TNFRSF10A-dependent anti-tumor activity in cholangiocarcinoma. While tumor infiltrating monocytes exhibited higher CSF1/M-CSF activity compared to peritumoral monocytes, they had lower *TRAIL*/*TNFSF10* expression. The strong negative prognostic value of the macrophage colony-stimulating factor CSF1/M-CSF in iCCA, with higher expression shown specifically in iCCA tumor-infiltrating T-cells, furthermore, implicates tumor associated macrophages as important actors in the promotion of tumor progression (24, 41). Additionally, an interplay between inflammatory factors and a local tumor promoting environment has been described in BTC (42, 43), with a role of myeloid-derived suppressor cells (41, 43). Finally, an anti-tumor activity of TRAIL/TNFSF10 in cholangiocarcinoma can also rely on additional mechanisms, namely activation of other TRAIL receptors than TRAIL-R1/TNFRSF10A, and targeting of other tumor promoting cells than just the tumor cells (44). In single cell analysis of iCCA, *TRAIL-R2*/*TNFRSF10B* was most highly expressed by endothelial cells but also expressed by tumor cells, immune cells, fibroblasts and cholangiocytes. A recent investigation of the iCCA T-cell and myeloid compartments exhibited agonistic TRAIL/TNFSF10 signaling as one significant interaction between regulatory T-cells and myeloid cells, where the TRAIL/TNFSF10-TRAIL-R2/TNFRSF10B interaction was most pronounced for dendritic cells (45). TRAIL-stimulation via TRAIL-R2/TNFRSF10B has been proposed to induce dendritic cell maturation rather than apoptosis (46). In both of the single-cell cohorts reported here, dendritic cells were the immune cells with the highest expression of *TRAIL-R2*/*TNFRSF10B*.

Further investigating the role of TRAIL in cholangiocarcinoma tumor tissue, there was an indication of a positive prognostic value in tissue expression of both *TRAIL-R2*/*TNFRSF10B* and *TRAIL-R4*/*TNFRSF10D* with a significant association to disease-free survival in the cohort from Ahn et al. (19) (GSE107943). This was furthermore supported by analysis of the cohort from Job et al. (24) (E-MTAB-6389) where *TRAIL-R1*/*TNFRSF10A* and *TRAIL-R4*/*TNFRSF10D* were significantly associated to overall survival. No association of *TRAIL-R* expression with survival was seen in the third iCCA cohort from Nakamura et al. (23), or in the mixed CCA cohorts from Andersen et al. (22) (GSE26566) and Jusakul et al. (21) (GSE89749). While disease-free survival was better for patients with high *TRAIL-R2*/*TNFRSF10B* and *TRAIL-R4*/*TNFRSF10D*, no significant association was seen between *TRAIL-R* expression and overall survival in the GSE107943 cohort from Ahn et al, possibly reflecting the low number of events and limited follow-up for the overall survival outcome (deaths = 17, median follow-up 30.5 months) (19).

In one iCCA cohort, the OEP001105 dataset reported by Dong et al. (25), *TRAIL-R* expression was instead negatively associated with survival. It has been established that cancer cells including CCA cell lines can develop resistance to TRAIL-induced apoptosis (47), with TRAIL-signaling instead contrarily inducing a tumor promoting inflammatory secretome, suggested to affect the tumor microenvironment (48). Underlying differences in tumor etiology and biology between the different investigated iCCA cohorts could also be one explanation to discrepancies in prognostic implications. Notably, in the Dong et al. cohort, the prevalence of underlying viral hepatitis was above 25% and approximately 10 percent of patients had a preoperative plasma AFP above 20 ng/mL (25). This AFP level has been used by a previous study as a cut off to exclude patients with possible mixed hepatocellular carcinoma-cholangiocarcinoma (HCC-CCA) (49). In the cohorts reported by Ahn et al. (19) (GSE107943) and Job et al. (24) (E-MTAB-6389) patients with HCC-CCA were excluded. It has been suggested that HCC cells can show considerable resistance to TRAIL-induced apoptosis (50), whereas no reports on this matter specific for HCC-CCA were found.

TRAIL-R4/TNFSRF10D, with a truncated intracellular death domain, can act as a decoy and antagonistic TRAIL receptor. However, in data from three cohorts, tumor tissue expression of *TRAIL-R4*/*TNFRSF10D* showed a similar prognostic influence as expression of the agonistic TRAIL receptors *TRAIL-R1*/*TNFRSF10A* and *TRAIL-R2*/*TNFRSF10B*: a congruent positive association to disease-free survival or overall survival in two cohorts, and to negative survival in one cohort. Distinctive TRAIL-signal responses in different cell types could be one possible explanation to such associations. As was the case with *TRAIL-R2*/*TNFRSF10B*, the highest *TRAIL-R4*/*TNFRSF10D* expression in iCCA was noted in endothelial cells.

Plasma TIE2/TEK, the angiopoietin receptor, was a strong negative prognostic factor for survival specifically in the GBC subgroup. Plasma TIE2 has been investigated as a biomarker during treatment with VEGFR inhibitor in advanced BTC (51). In the tumor micro-environment of several cancers, a subset of TIE2-expressing tumor associated macrophages has been described, with proangiogenic activity and negative prognostic value (52), implicating the interplay between tumor associated macrophages and angiogenesis as a possible therapeutic target (53). In an analysis of TIE2-expressing tumor associated macrophages in pCCA, a positive association to survival was instead found (54). As opposed to some other types of highly vascularized malignancies, CCA tissues can be characterized by a dense fibrous stroma (4, 54). Whereas VEGFR inhibition alone has failed to show improved outcomes in BTC, a targeted combined inhibition of VEGFR and TIE2 recently showed a significant effect on progression-free survival of BTC in a phase two randomized control trial (55). Importantly, the vascular endothelium can have several roles, not only with regards to tumor angiogenesis but also in the regulation of immune cell infiltration and itself acting as a regulator of immune cell function (56).

While prognostic associations of soluble factors in plasma may reflect mechanistic processes in tumor and peritumoral tissue, it is also possible that the plasma protein profile reflects a systemic host response to malignancy or concurrent inflammatory conditions. Two of the identified prognostic markers, CSF1/M-CSF and TIE2/TEK, were differentially expressed in malignancy compared to benign controls. This was also the case with IL6, which in this study showed a univariable association to survival in resected patients and previously has been validated as a prognostic factor in advanced BTC (11), with mixed previous reports on possible diagnostic value (57, 58). Levels of differentially expressed proteins overlapped between the malignant and benign groups, with predictive value for the highest expression levels, but low sensitivity. While beyond the scope of this study, the possible diagnostic value of CSF1/M-CSF and PGF in combination with other factors should be investigated in specialized diagnostic studies. To clarify the role of infiltrating immune cells and the tumor microenvironment on one hand, and the systemic inflammatory response in BTC on the other, further analyses of CCA and GBC tissue, including single-cell and spatial transcriptomics and histopathology, are motivated. That no proteins were significantly differentially expressed between patients undergoing resection and patients with unresectable tumors could reflect that patients with resectable (localized) and unresectable (advanced/metastasized) tumors represent a spectrum of disease rather than clear-cut separated categories. Indeed, in pancreatic cancer, a malignancy with similarly poor long-term prognosis, patients with localized tumors undergoing resection have been found to harbor distant micrometastases (59, 60). Secondly, the small sample-size with only 27 patients with unresectable tumors limited the statistical power of this study to detect a significant difference in expression between patients with resectable and unresectable BTC.

An important strength of the current study was a dedicated prospective research biobank allowing the inclusion of a comparatively large cohort of patients resected for BTC, a group of rare cancers most often diagnosed at an unresectable stage. Furthermore, patients were followed for a median time of more than five years after surgery, allowing an accurate analysis of long-term survival. Other strengths include the method for relative quantification of protein expression by multiplexed immunoassay with strong internal quality controls minimizing variability. Finally, the findings from the plasma biomarker screening were put in a comprehensive context with analysis of tissue gene expression for markers and receptors/ligands in both tumor and surrounding liver tissue from patients with BTC in demographically varied cohorts.

The study also had several important limitations. Firstly, the sample size was limited and calculated to allow the identification of a prognostic marker for patients with BTC of any subtype. With differences in prognostic value seen between BTC subgroups, most importantly for TIE2, a larger sample size would have permitted further analyses and reduced the risk of error and overfitting. While inclusion and sample collection in the biobank were prospective, collection of clinical follow-up data was retrospective, and no further postoperative biobank samples were included in the protocol precluding analysis of temporal dynamics in biomarker expression. Furthermore, while prognostic associations for the bulk tissue expression of markers and receptors/ligands in external cohorts was studied in all subgroups of BTC, single cell analysis was limited to the iCCA subgroup.

In conclusion, with this analysis of a unique prospectively collected biobank three preoperative prognostic factors could be identified in plasma from patients with BTC, with plasma TRAIL/TNFSF10 determined as a novel positive prognostic factor in both iCCA and pCCA. With subgroup analyses and interrogation of external cohorts, the heterogeneity both between and within BTC subgroups was underscored, a factor of vital importance when developing future targeted treatments. A negative prognostic value of plasma CSF1/M-CSF was seen in iCCA and GBC, further implicating tumor-associated macrophages and the interplay between inflammatory activity and tumor progression as a possible therapeutic target in BTC. TRAIL and CSF1, both prognostic factors in iCCA, exhibited marked differences in expression and activity between intratumoral and peritumoral immune cells on single-cell analysis. The negative prognostic value of plasma TIE2/TEK in GBC mandates further investigation of proangiogenic and inflammatory activity in GBC tumor tissue. Validation of predictive value in external and prospective cohorts will be the next step in the development of disease-specific preoperative prognostic models for patients with BTC.

## Data availability statement

The raw data supporting the conclusions of this article will be made available by the authors, without undue reservation.

## Ethics statement

This study involving human participants was reviewed and approved by the Regional Ethical Review Board of Stockholm. All patients included in the biobank provided their written informed consent.

## Author contributions

Conceptualization: HJ, MC, CS, NB, ES. Methodology: all authors. Investigation: HJ, MC, DS, IF, CO’R, JA, NB, ES. Writing – original draft: HJ, DS, MC, NB, ES. Writing – review and editing: all authors. Funding acquisition: HJ, MC, NB, ES. Resources: JA, NB, ES. Supervision: MC, NB, ES. All authors contributed to the article and approved the submitted version.
