# Erratum: Preoperative immunological plasma markers TRAIL, CSF1, and TIE2 predict survival after resection for biliary tract cancer

**DOI:** 10.3389/fonc.2023.1281693

**Published:** 2023-09-27

**Authors:** Frontiers Production Office

**Affiliations:** Frontiers Media SA, Lausanne, Switzerland

**Keywords:** cholangiocarcinoma (CCA), gallbladder cancer (GBC), prognostic biomarkers, tumor associated macrophage (TAM), biliary tract cancer (BTC)

Due to a production error, there was a mistake in the legend for **Figure 1** as published. The term “pCCA” was incorrectly formatted as “PCCA”. The correct legend appears below. The publisher apologizes for this mistake.

**Figure 1:**
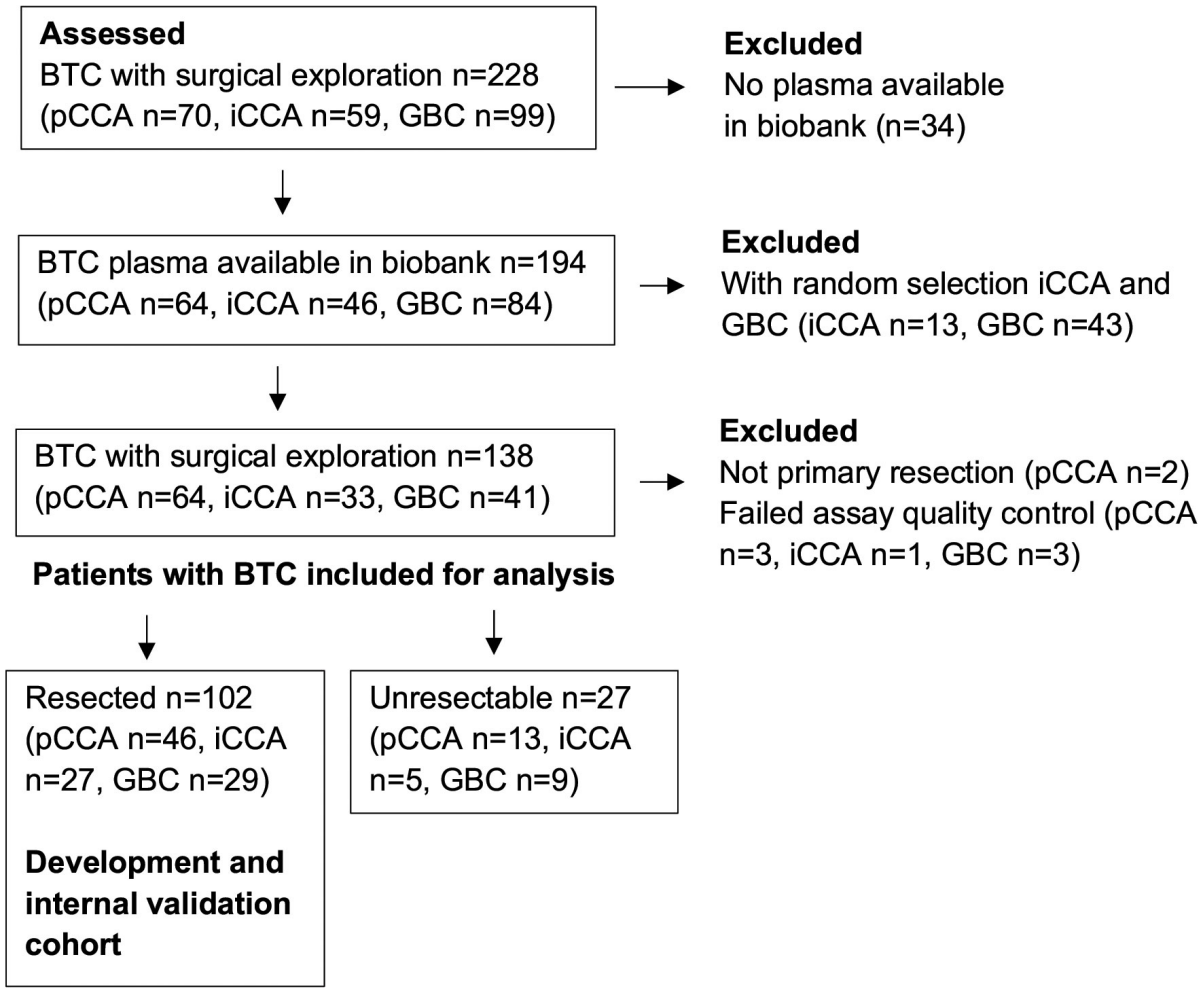
Study flow chart for the inclusion of patients with BTC. BTC, biliary tract cancer; GBC, gallbladder cancer; iCCA, intrahepatic cholangiocarcinoma; pCCA, perihilar cholangiocarcinoma.

The original version of this article has been updated.

