# Supplementary material for: Preoperative immunological plasma markers TRAIL, CSF1 and TIE2 predict survival after resection for biliary tract cancer
Source: Front Oncol. 2023 Jun 19;13:1169537. doi: 10.3389/fonc.2023.1169537 (PMC10315823; doi:10.3389/fonc.2023.1169537)
Supplement: Supplementary file 1 [file DataSheet_1.pdf]

Supplementary Material

Preoperative immunological plasma markers TRAIL, CSF1 and TIE2 predict survival after resection for biliary tract cancer

Hannes Jansson[1*], Martin Cornillet[2], Dan Sun[2], Iva Filipovic[2], Christian Sturesson[1], Colm J. O'Rourke[3], Jesper B. Andersen[3], Niklas K. Björkström[2†], Ernesto Sparrelid[2†]

[1]Division of Surgery and Oncology, Department of Clinical Science, Intervention and Technology, Karolinska Institutet, Karolinska University Hospital, Stockholm, Sweden

[2]Center for Infectious Medicine, Department of Medicine Huddinge, Karolinska Institutet, Karolinska University Hospital, Stockholm, Sweden

[3]Biotech Research and Innovation Centre (BRIC), Department of Health and Medical Sciences, University of Copenhagen, Copenhagen, Denmark

*** Correspondence:**
Hannes Jansson
hannes.jansson@ki.se

† These authors contributed equally to this work and share senior authorship

1 Supplementary Figures and Tables

1.1 Supplementary Figures

1.1.1 Supplementary Figure 1

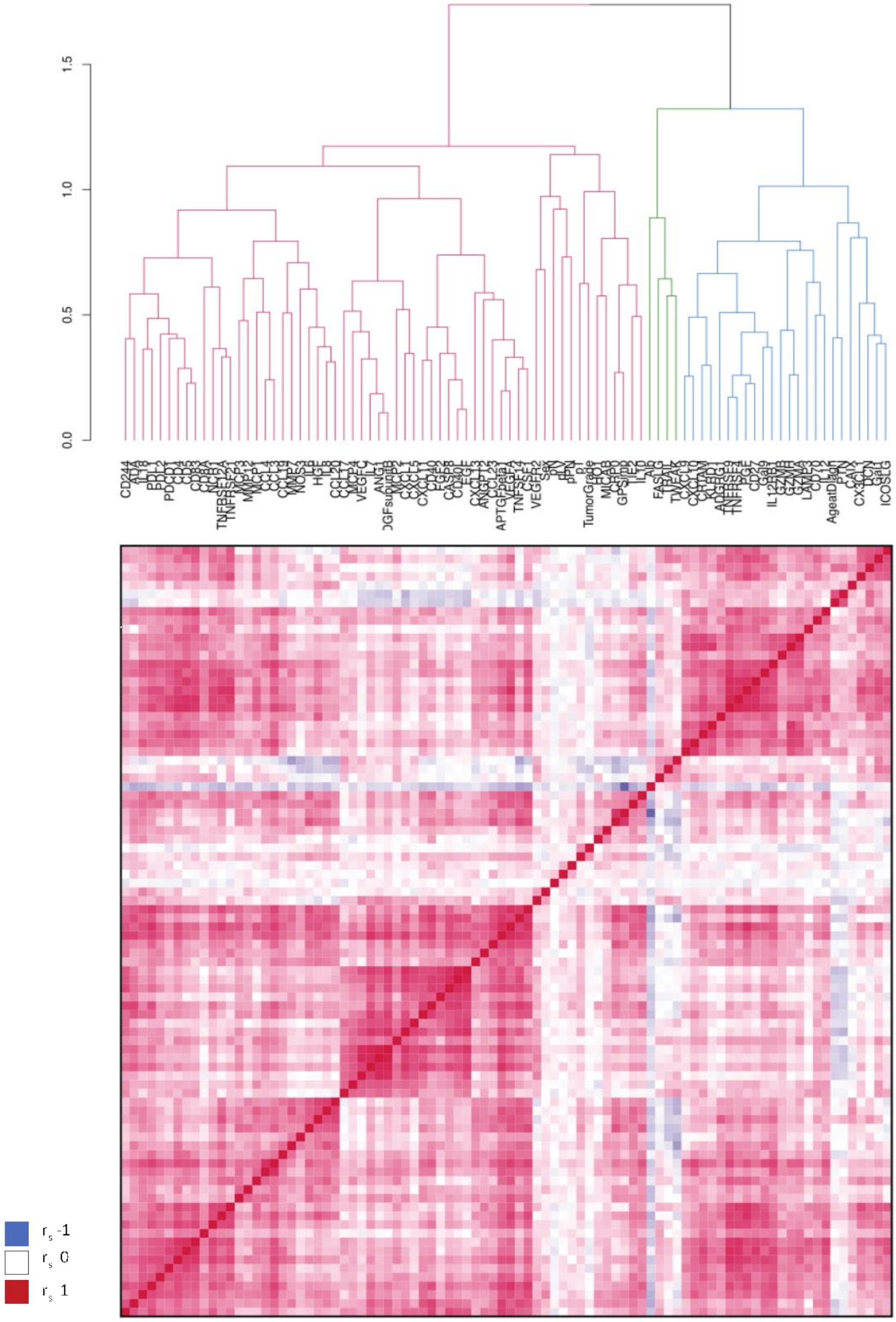

pT: tumor extension, pN: lymph node metastasis, pPN: perineural invasion, pLV: lympho-vascular invasion, AgeatDiagn: age, CRP0: preoperative CRP, Alb: preoperative albumin; GPSimp: Glasgow prognostic score, TumorGrade: tumor grade according to the College of American Pathologists.

Supplementary Figure 1. Correlation matrix including clinicopathological variables

1.1.2 Supplementary Figure 2

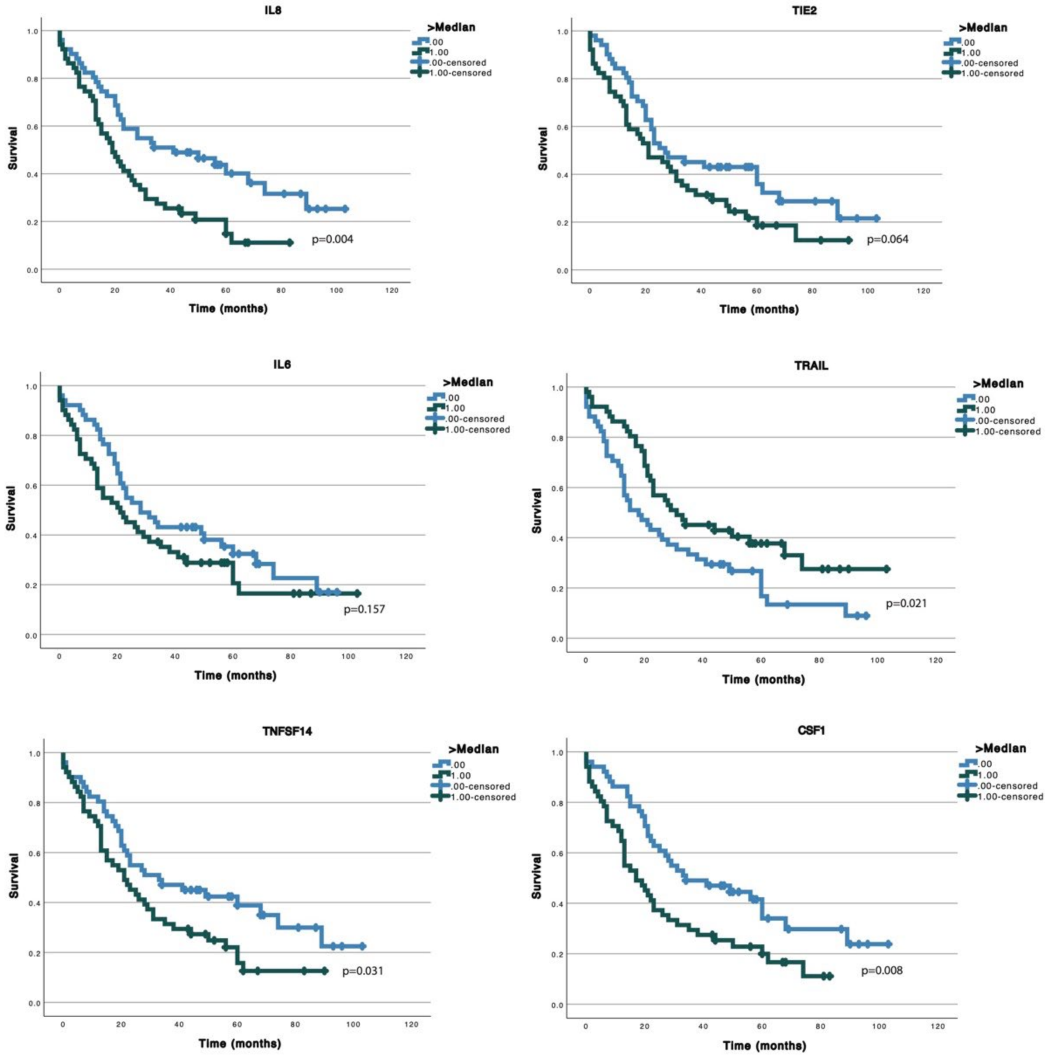

Supplementary Figure 2. Kaplan-Meier survival estimates according to marker plasma expression (Normalized Protein Expression [NPX] units, above/below median)

1.1.3 Supplementary Figure 3

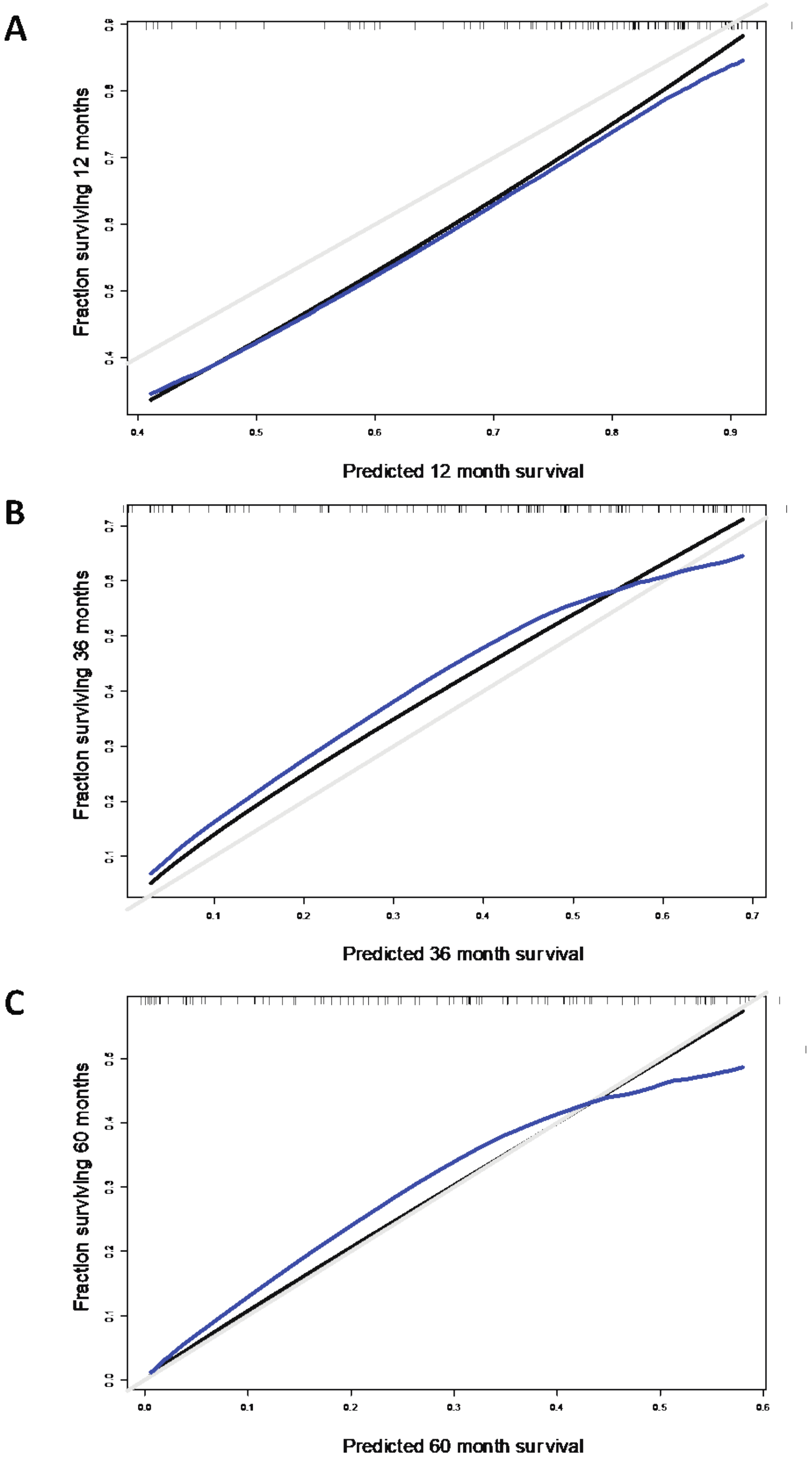

Supplementary Figure 3: Calibration of a preoperative prognostic model (TRAIL, CSF1, TIE2) for survival at 1 year **(A)**, 3 years **(B)** and 5 years **(C)**

1.1.4 Supplementary Figure 4

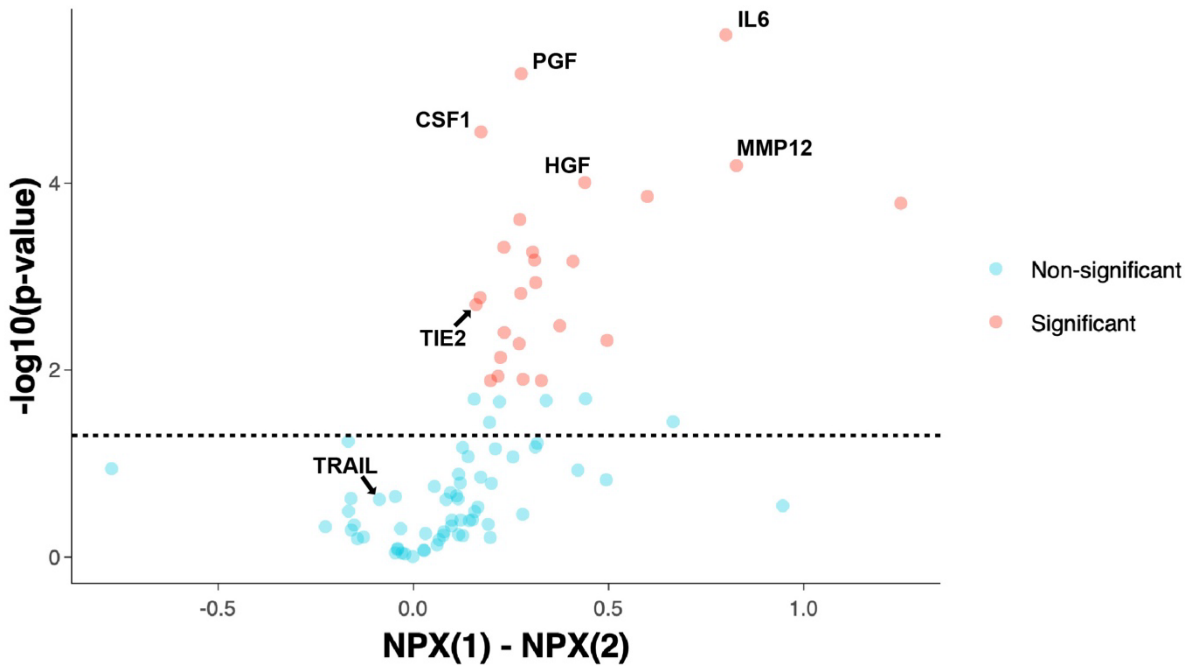

BTC: biliary tract cancer; NPX: normalized protein expression

Supplementary Figure 4: Differential expression of plasma proteins between patients with BTC (1) and benign histopathology (2)

1.1.5 Supplementary Figure 5

A

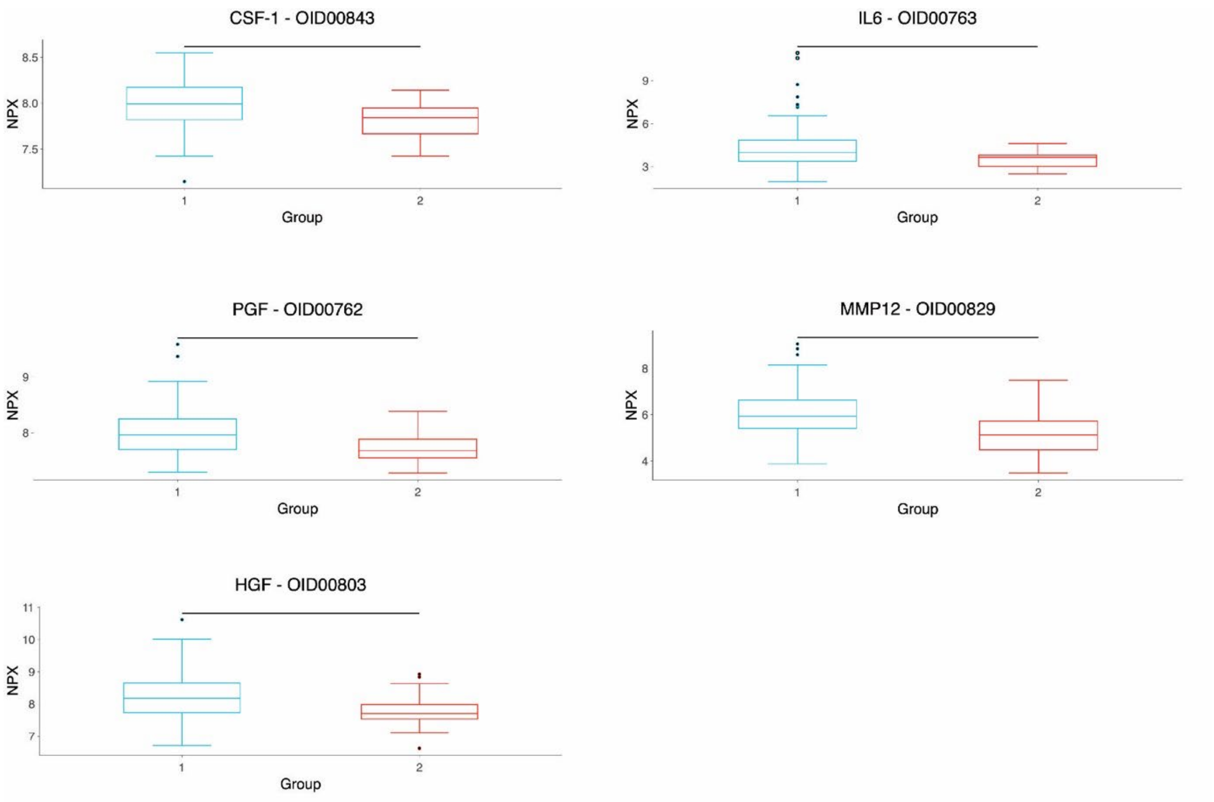

B

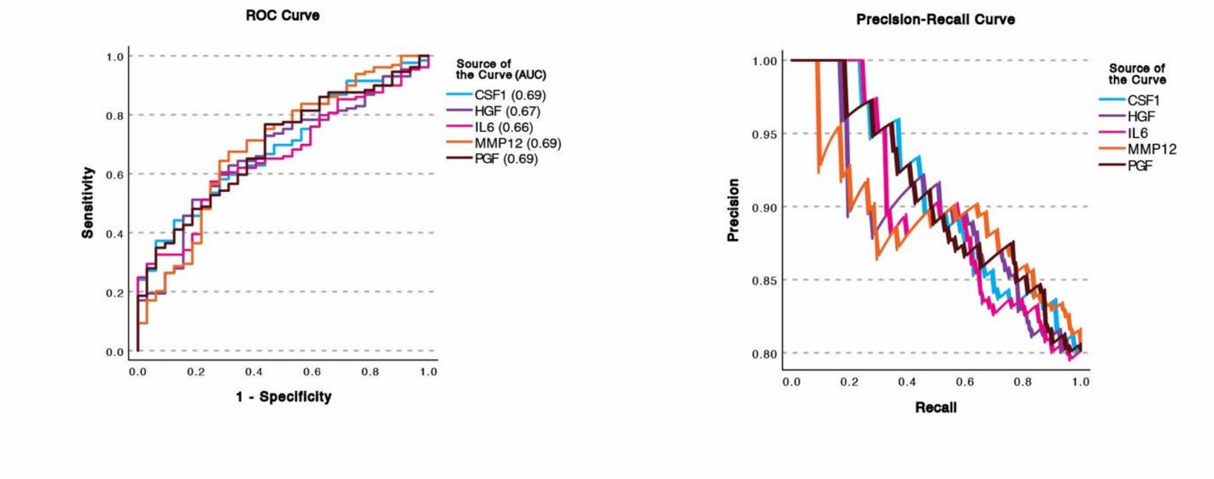

AUROC: area under the receiver-operating curve; BTC: biliary tract cancer; NPX: normalized protein expression; OID: Olink assay identification; ROC: receiver-operating curve

Supplementary Figure 5: Overlap of markers in BTC and benign conditions **(A)**, AUROC and precision-recall analyses **(B)**

1.1.6 **Supplementary Figure 6A-B**

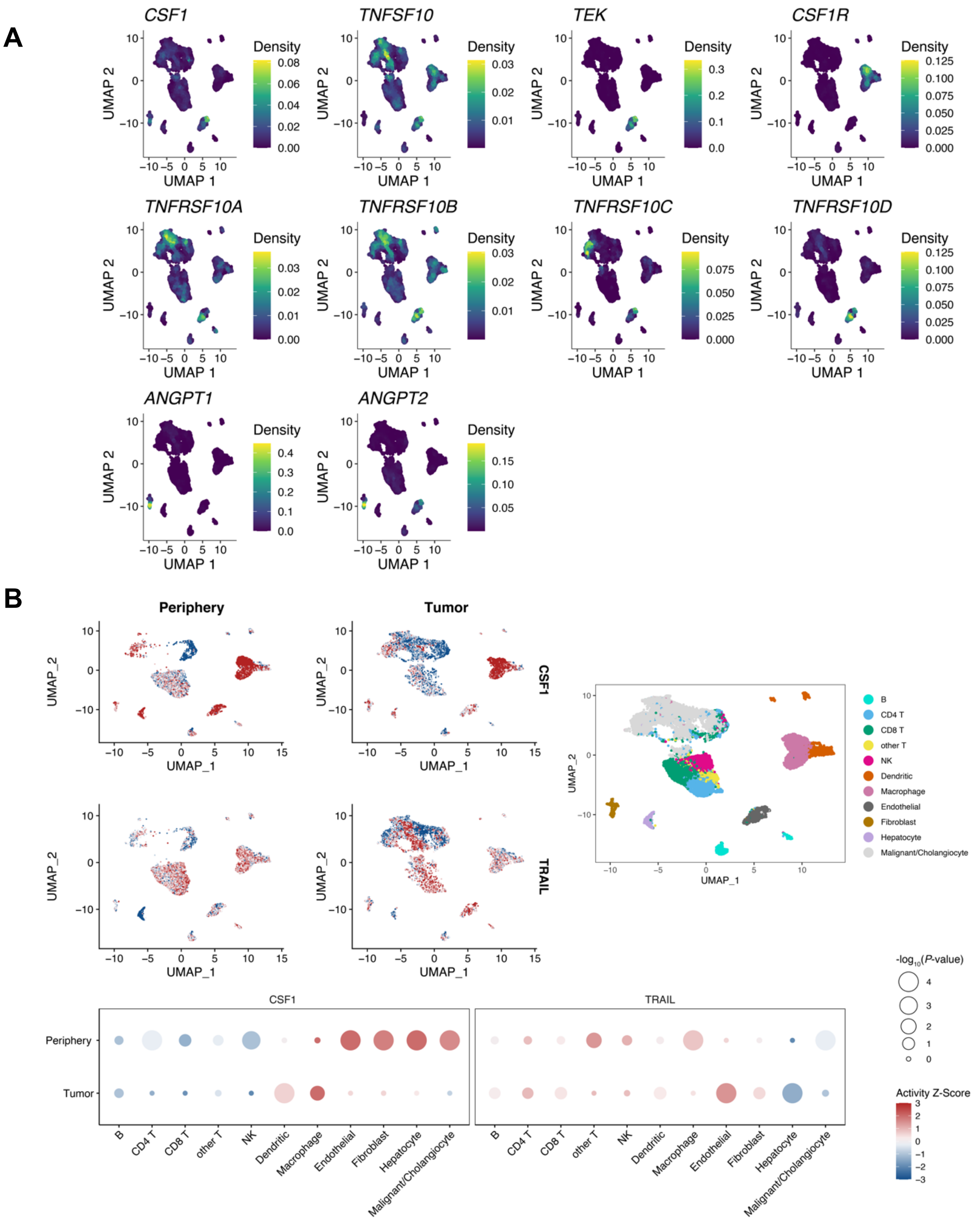

B: B-lymphocyte; iCCA: intrahepatic cholangiocarcinoma; NK: natural killer cell; T: T-lymphocyte; UMAP: unified manifold apporximation and projection.

Supplementary Figure 6A-B: Single-cell gene expression of markers and receptors/ligands in iCCA (Zhang et al.), clusters by cell type (left top panel **B**). **B:** Cytokine activity intratumorally vs. peritumorally. Activity Z-scores were trimmed to [-3, 3] to facilitate visualization. P-values in balloon plots were calculated by permutation tests (see Material and Methods)

1.1.7 Supplementary Figure 7

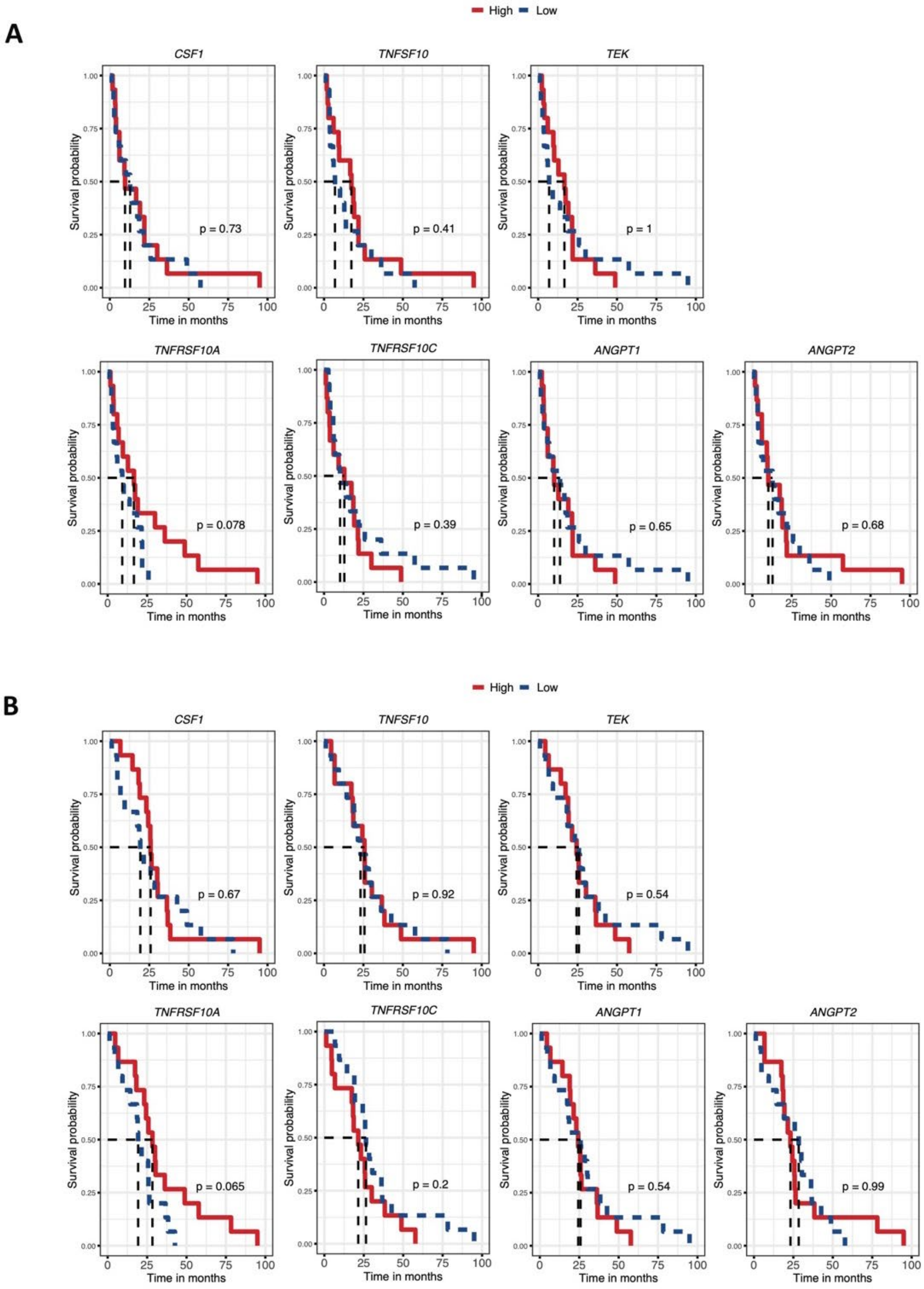

Supplementary Figure 7: Analysis of association of iCCA tumor tissue gene expression of markers and receptors/ligands, with disease-free survival **(A)** and overall survival **(B)** (Ahn et al.)

1.1.8 Supplementary Figure 8

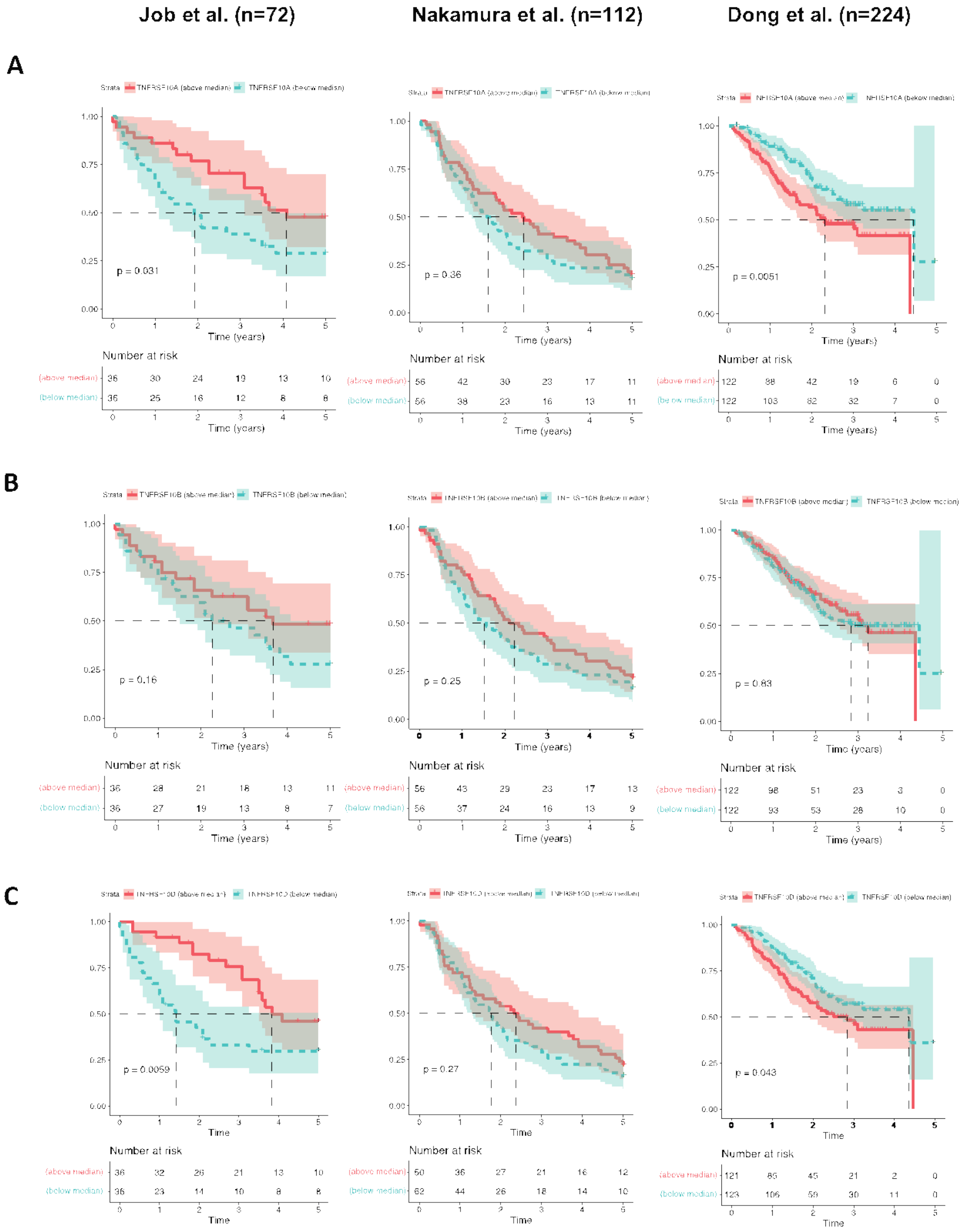

iCCA: intrahepatic cholangiocarcinoma

Supplementary Figure 8: Prognostic influence of tumor tissue expression of TRAIL receptors in iCCA, *TRAILR1/TNFRSF10A* **(A)**; *TRAILR2/TNFRSF10B* **(B)**; *TRAILR4/TNFRSF10D* **(C)**

1.2 Supplementary Tables

1.2.1 Supplementary Table 1: REMARK checklist

	Item to be reported	Page no.
**INTRODUCTION**		
1	State the marker examined, the study objectives, and any pre-specified hypotheses.	2
**MATERIALS AND METHODS**		
Patients		
2	Describe the characteristics (e.g., disease stage or co-morbidities) of the study patients, including their source and inclusion and exclusion criteria.	3
3	Describe treatments received and how chosen (e.g., randomized or rule-based).	3
Specimen characteristics		
4	Describe type of biological material used (including control samples) and methods of preservation and storage.	3-4
Assay methods		
5	Specify the assay method used and provide (or reference) a detailed protocol, including specific reagents or kits used, quality control procedures, reproducibility assessments, quantitation methods, and scoring and reporting protocols. Specify whether and how assays were performed blinded to the study endpoint.	3-4
Study design		
6	State the method of case selection, including whether prospective or retrospective and whether stratification or matching (e.g., by stage of disease or age) was used. Specify the time period from which cases were taken, the end of the follow-up period, and the median follow-up time.	3-5
7	Precisely define all clinical endpoints examined.	4
8	List all candidate variables initially examined or considered for inclusion in models.	3, Supplementary Table 2
9	Give rationale for sample size; if the study was designed to detect a specified effect size, give the target power and effect size.	3
Statistical analysis methods		
10	Specify all statistical methods, including details of any variable selection procedures and other model-building issues, how model assumptions were verified, and how missing data were handled.	5
11	Clarify how marker values were handled in the analyses; if relevant, describe methods used for cutpoint determination.	3-5
**RESULTS**		
Data		
12	Describe the flow of patients through the study, including the number of patients included in each stage of the analysis (a diagram may be helpful) and reasons for dropout. Specifically, both overall and for each subgroup extensively examined report the numbers of patients and the number of events.	3, Figure 1
13	Report distributions of basic demographic characteristics (at least age and sex), standard (disease-specific) prognostic variables, and tumor marker, including numbers of missing values.	Table 1
Analysis and presentation		
14	Show the relation of the marker to standard prognostic variables.	Supplementary Figure 1
15	Present univariable analyses showing the relation between the marker and outcome, with the estimated effect (e.g., hazard ratio and survival probability). Preferably provide similar analyses for all other variables being analyzed. For the effect of a tumor marker on a time-to-event outcome, a Kaplan-Meier plot is recommended.	Supplementary Table 6-7
16	For key multivariable analyses, report estimated effects (e.g., hazard ratio) with confidence intervals for the marker and, at least for the final model, all other variables in the model.	Table 2
17	Among reported results, provide estimated effects with confidence intervals from an analysis in which the marker and standard prognostic variables are included, regardless of their statistical significance.	Supplementary Table 7
18	If done, report results of further investigations, such as checking assumptions, sensitivity analyses, and internal validation.	6-7
**DISCUSSION**		
19	Interpret the results in the context of the pre-specified hypotheses and other relevant studies; include a discussion of limitations of the study.	10-13
20	Discuss implications for future research and clinical value.	2, 12-13

Source: McShane LM, Altman DG, Sauerbrei W, Taube SE, Gion M, Clark GM: Reporting recommendations for tumor marker prognostic studies (REMARK). *J Natl Cancer Inst* 2005; 97: 1180-1184.

1.2.2 Supplementary Table 2

IL8 (P10145)	Gal1 (P09382)
TNFRSF9 (Q07011)	PDL1 (Q9NZQ7)
TIE2 (Q02763)	CD27 (P26842)
MCP3 (P80098)	CXCL5 (P42830)
CD40L (P29965)	IL5 (P05113)
IL1alpha (P01583)	HGF (P14210)
CD244 (Q9BZW8)	GZMA (P12544)
EGF (P01133)	HO1 (P09601)
ANG1 (Q15389)	CX3CL1 (P78423)
IL7 (P13232)	CXCL10 (P02778)
PGF (P49763)	CD70 (P32970)
IL6 (P05231)	IL10 (P22301)
ADGRG1 (Q9Y653)	TNFRSF12A (Q9NP84)
MCP1 (P13500)	CCL23 (P55773)
CRTAM (O95727)	CD5 (P06127)
CXCL11 (O14625)	CCL3 (P10147)
MCP4 (Q99616)	MMP7 (P09237)
TRAIL (P50591)	ARG1 (P05089)
FGF2 (P09038)	NCR1 (O76036)
CXCL9 (Q07325)	DCN (P07585)
CD8A (P01732)	TNFRSF21 (O75509)
CAIX (Q16790)	TNFRSF4 (P43489))
IFNbeta (P01574)	MICAB (Q29983/Q29980)
ADA (P00813)	CCL17 (Q92583)
CD4 (P01730)	ANGPT2 (O15123)
NOS3 (P29474)	PTN (P21246)
IL2 (P60568)	CXCL12 (P48061)
Gal9 (O00182)	IFNgamma (P01579)
VEGFR2 (P35968)	LAMP3 (Q9UQV4)
CD40 (P25942)	CASP8 (Q14790)
IL18 (Q14116)	ICOSLG (O75144)
GZMH (P20718)	MMP12 (P39900)
VEGFC (P49767)	CXCL13 (O43927)
LAPTGFbeta1 (P01137)	PDL2 (Q9BQ51)
CXCL1 (P09341)	VEGFA (P15692)
TNFSF14 (O43557)	IL4 (P05112)
IL33 (O95760)	IL21 (Q9HBE4)
TWEAK (O43508)	IL12RB1 (P42701)
PDGFsubunitB (P01127)	IL13 (P35225)
PDCD1 (Q15116)	CCL20 (P78556)
FASLG (P48023)	TNF (P01375)
CD28 (P10747)	KLRD1 (Q13241)
CCL19 (Q99731)	GZMB (P10144)
MCP2 (P80075)	CD83 (Q01151)
CCL4 (P13236)	IL12 (P29459/P29460)
IL35 (Q14213)	CSF1 (P09603)

Supplementary Table 2: Panel of analytes for proximity extension assay (UniProt ID)

1.2.3 Supplementary Table 3

IL8 (P10145)	CCL4 (P13236)
TNFRSF9 (Q07011)	Gal1 (P09382)
TIE2 (Q02763)	PDL1 (Q9NZQ7)
MCP3 (P80098)	CD27 (P26842)
CD40L (P29965)	CXCL5 (P42830)
CD244 (Q9BZW8)	HGF (P14210)
EGF (P01133)	GZMA (P12544)
ANG1 (Q15389)	HO1 (P09601)
IL7 (P13232)	CX3CL1 (P78423)
PGF (P49763)	CXCL10 (P02778)
IL6 (P05231)	CD70 (P32970)
ADGRG1 (Q9Y653)	IL10 (P22301)
MCP1 (P13500)	TNFRSF12A (Q9NP84)
CRTAM (O95727)	CCL23 (P55773)
CXCL11 (O14625)	CD5 (P06127)
MCP4 (Q99616)	CCL3 (P10147)
TRAIL (P50591)	MMP7 (P09237)
FGF2 (P09038)	NCR1 (O76036)
CXCL9 (Q07325)	DCN (P07585)
CD8A (P01732)	TNFRSF21 (O75509)
CAIX (Q16790)	TNFRSF4 (P43489)
ADA (P00813)	MICAB (Q29983/Q29980)
CD4 (P01730)	CCL17 (Q92583)
NOS3 (P29474)	ANGPT2 (O15123)
Gal9 (O00182)	PTN (P21246)
VEGFR2 (P35968)	LAMP3 (Q9UQV4)
CD40 (P25942)	CASP8 (Q14790)
IL18 (Q14116)	ICOSLG (O75144)
GZMH (P20718)	MMP12 (P39900)
VEGFC (P49767)	CXCL13 (O43927)
LAPTGFbeta1 (P01137)	PDL2 (Q9BQ51)
CXCL1 (P09341)	VEGFA (P15692)
TNFSF14 (O43557)	IL12RB1 (P42701)
TWEAK (O43508)	CCL20 (P78556)
PDGFsubunitB (P01127)	KLRD1 (Q13241)
PDCD1 (Q15116)	GZMB (P10144)
FASLG (P48023)	CD83 (Q01151)
CCL19 (Q99731)	IL12 (P29459/P29460)
MCP2 (P80075)	CSF1 (P09603)

Supplementary Table 3: Proteins detected by proximity extension assay in >75% of plasma samples (UniProt ID)

1.2.4 Supplementary Table 4

Protein	Unresectable BTC (mean NPX)	Resected BTC (mean NPX)	p-value (adjusted)$
CSF-1	8.12074259259259	7.95936647058824	0.195
HGF	8.56709851851852	8.13772196078431	0.195
TNFSF14	5.00247740740741	4.48406950980392	0.195
TNFRSF12A	6.44771666666667	6.13527049019608	0.195
LAP TGF-beta-1	1.99445518518519	1.71597980392157	0.195
VEGFA	8.7554162962963	8.42750539215686	0.195
CCL3	6.47316444444444	6.05065921568627	0.195
ADA	3.19125851851852	2.94662117647059	0.195
IL6	5.06665814814815	4.11963862745098	0.195
CASP-8	4.49502555555556	4.16187568627451	0.195
CD4	1.26537037037037	1.06110754901961	0.195
TNFRSF4	3.89925296296296	3.61926539215686	0.197
PGF	8.19746851851852	7.9564768627451	0.208
NOS3	1.43487538461538	1.10347918367347	0.208
TNFRSF9	6.19355037037037	5.88118068627451	0.208
EGF	8.44440481481482	7.90452598039216	0.208
TNFRSF21	7.97091037037037	7.83932558823529	0.208
CCL4	7.82080592592593	7.47915598039216	0.208
ADGRG1	2.16807222222222	1.6475133	0.208
MMP12	6.40588037037037	5.91144225490196	0.208
CXCL11	7.00922444444444	6.4073718627451	0.224
FASLG	5.32417851851852	5.53959401960784	0.224
PD-L1	4.99091666666667	4.75185431372549	0.224
CD27	7.92470777777778	7.69948568627451	0.224
CD244	6.08397666666667	5.89371303921569	0.224
CD40-L	6.66632851851852	6.18571823529412	0.224
Gal-9	7.9392762962963	7.7768881372549	0.224
CD83	2.80845518518519	2.59282441176471	0.224
IL12	7.07872185185185	6.82476039215686	0.238
TIE2	7.68045777777778	7.55970676470588	0.238
CX3CL1	5.86808888888889	5.69622343137255	0.238
CCL19	10.466287037037	10.128236372549	0.238
TRAIL	7.57525037037037	7.74886794117647	0.238
CAIX	5.25308555555556	4.77291852941176	0.238
IL10	3.88134037037037	3.36722392156863	0.238
IL7	5.26055074074074	4.97488254901961	0.266
IL8	7.56978148148148	7.14341843137255	0.270
IL12RB1	2.02893592592593	1.84255245098039	0.271
CCL23	10.7636407407407	10.5029554901961	0.271
CD40	11.7055548148148	11.519852745098	0.273
TWEAK	8.01229296296296	8.1392118627451	0.273
MCP-3	2.31818296296296	1.97607235294118	0.273
CCL20	6.85226703703704	6.2671112745098	0.273
MMP7	9.7573537037037	9.60974156862745	0.279
CD5	4.80568740740741	4.68426352941176	0.318
PDCD1	3.05080037037037	2.84561460784314	0.318
NCR1	3.17913444444444	3.0214262745098	0.357
MCP-2	7.15751925925926	6.99642294117647	0.364
ANGPT2	5.53771259259259	5.39685607843137	0.389
CXCL1	9.79487074074074	9.55489735294118	0.390
CXCL13	8.97076777777778	8.77301490196079	0.402
Gal-1	6.38950888888889	6.32556264705882	0.420
PD-L2	2.29558962962963	2.17309039215686	0.447
MCP-4	6.6513162962963	6.48914049019608	0.454
CRTAM	4.71928962962963	4.57868764705882	0.454
MCP-1	10.8139974074074	10.6601298039216	0.466
HO-1	12.0438692592593	12.1870069607843	0.466
DCN	4.63591	4.56571078431373	0.480
VEGFR-2	6.63683037037037	6.67977754901961	0.527
CXCL10	8.16256555555556	7.90493480392157	0.527
FGF2	1.42054230769231	1.25167540816327	0.532
PTN	4.56516296296296	4.29304411764706	0.549
ICOSLG	4.94806222222222	4.89475009803922	0.549
IL18	9.10512037037037	8.98837666666667	0.585
VEGFC	0.757464814814815	0.619212352941176	0.611
PDGF subunit B	10.1682181481481	10.0177464705882	0.625

CD70	3.15278518518519	3.08575303921569	0.651
ANG-1	8.37114296296296	8.25471166666667	0.732
GZMB	3.36820296296296	3.45463647058824	0.737
CXCL5	11.2904433333333	11.4125151960784	0.740
CD8A	9.66248481481482	9.72082735294118	0.821
LAMP3	4.55843	4.51782049019608	0.847
CCL17	7.19590962962963	7.26537303921569	0.847
GZMA	5.57923703703704	5.61182950980392	0.847
MIC-A/B	3.91283444444444	3.99581450980392	0.879
CXCL9	8.23976888888889	8.19924470588235	0.879
GZMH	5.22546	5.26158068627451	0.889
KLRD1	6.1567137037037	6.16814715686275	0.940

BTC: biliary tract cancer; NPX: normalized protein expression

$ Benjamini-Hochberg method

Supplementary Table 4: Differential expression between patients resected for BTC and patients with unresectable BTC

1.2.5 Supplementary Table 5

**CLUSTER 1**	
VEGFR2 (P35968)	
FASLG (P48023)	
TRAIL (P50591)	
TWEAK (O43508)	
**CLUSTER 2**	
CCL17 (Q92583)	HO1 (P09601)
MCP4 (Q99616)	MICAB (Q29983/Q29980)
VEGFC (P49767)	CD244 (Q9BZW8)
IL7 (P13232)	ADA (P00813)
ANG1 (Q15389)	IL18 (Q14116)
PDGFsubunitB (P01127)	PDL1 (Q9NZQ7)
MCP2 (P80075)	PDL2 (Q9BQ51)
CXCL1 (P09341)	PDCD1 (Q15116)
CXCL5 (P42830)	CD4 (P01730)
CXCL11 (O14625)	CD5 (P06127)
CD40 (P25942)	CD83 (Q01151)
FGF2 (P09038)	CD8A (P01732)
CASP8 (Q14790)	NCR1 (O76036)
CD40L (P29965)	TNFRSF12A (Q9NP84)
EGF (P01133)	TNFRSF21 (O75509)
CXCL13 (O43927)	NOS3 (P29474)
ANGPT2 (O15123)	IL6 (P05231)
CCL23 (P55773)	HGF (P14210)
LAPTGFbeta1 (P01137)	IL8 (P10145)
VEGFA (P15692)	CCL20 (P78556)
TNFSF14 (O43557)	MCP3 (P80098)
CSF1 (P09603)	MMP12 (P39900)
	MCP1 (P13500)
CXCL9 (Q07325)	CCL4 (P13236)
CXCL10 (P02778)	CCL3 (P10147)
CRTAM (O95727)	TIE2 (Q02763)
KLRD1 (Q13241)	IL10 (P22301)
ADGRG1 (Q9Y653)	CCL19 (Q99731)
TNFRSF9 (Q07011)	MMP7 (P09237)
TNFRSF4 (P43489)	
PGF (P49763)	
CD27 (P26842)	
Gal9 (O00182)	
IL12RB1 (P42701)	
GZMB (P10144)	
GZMH (P20718)	
GZMA (P12544)	
LAMP3 (Q9UQV4)	
CD70 (P32970)	
IL12 (P29459/P29460)	
CX3CL1 (P78423)	
DCN (P07585)	
Gal1 (P09382)	
ICOSLG (O75144)	
CAIX (Q16790)	
PTN (P21246)	

Supplementary Table 5: Proteins (UniProt ID) grouped by hierarchical clustering

1.2.6 Supplementary Table 6

Protein	HR (95% CI)	p-value	p-value (adjusted)$
IL8	1.3 (1.1-1.6)	0.00074	0.05550
TNFRSF9	1.1 (0.66-1.7)	0.82	1.00000
TIE2	4.5 (2-9.9)	0.00021	0.01596
MCP3	1.4 (1-1.8)	0.021	1.00000
CD40L	1.2 (0.98-1.4)	0.085	1.00000
CD244	1.2 (0.71-2)	0.51	1.00000
EGF	1.2 (1-1.5)	0.055	1.00000
ANG1	1.1 (0.86-1.4)	0.5	1.00000
IL7	1.1 (0.83-1.4)	0.57	1.00000
PGF	1.5 (0.81-3)	0.19	1.00000
IL6	1.3 (1.1-1.6)	0.0021	0.15330
ADGRG1	1 (0.8-1.3)	0.93	1.00000
MCP1	1.1 (0.72-1.6)	0.7	1.00000
CRTAM	1.2 (0.81-1.7)	0.43	1.00000
CXCL11	1.1 (0.97-1.3)	0.12	1.00000
MCP4	1 (0.76-1.4)	0.84	1.00000
TRAIL	0.34 (0.18-0.66)	0.0013	0.09620
FGF2	1.4 (1.1-1.8)	0.014	1.00000
CXCL9	0.89 (0.69-1.1)	0.36	1.00000
CD8A	1.3 (0.91-1.7)	0.17	1.00000
CAIX	1.3 (1.1-1.6)	0.013	1.00000
ADA	1.3 (0.78-2.1)	0.32	1.00000
CD4	1.1 (0.61-2.1)	0.68	1.00000
NOS3	1.2 (0.89-1.7)	0.2	1.00000
Gal9	1.3 (0.76-2.3)	0.32	1.00000
VEGFR2	1 (0.42-2.5)	0.95	1.00000
CD40	1.4 (0.94-2)	0.099	1.00000
IL18	1.4 (1-1.8)	0.042	1.00000
GZMH	1.1 (0.83-1.4)	0.53	1.00000
VEGFC	1.1 (0.82-1.4)	0.62	1.00000
LAPTGFbeta1	1.7 (1-3)	0.045	1.00000
CXCL1	1.4 (1-1.8)	0.03	1.00000
TNFSF14	1.9 (1.3-2.6)	2e-04	0.01540
TWEAK	0.58 (0.33-1)	0.059	0.96600
PDGFsubunitB	1.2 (0.95-1.6)	0.11	1.00000
PDCD1	1.1 (0.77-1.6)	0.6	1.00000
FASLG	0.63 (0.36-1.1)	0.095	0.91000
CCL19	0.89 (0.65-1.2)	0.45	1.00000
MCP2	0.84 (0.58-1.2)	0.35	1.00000
CCL4	1.1 (0.76-1.7)	0.54	1.00000
Gal1	1 (0.41-2.4)	1	1.00000
PDL1	1.1 (0.65-1.8)	0.79	1.00000
CD27	1.1 (0.63-1.8)	0.85	1.00000
CXCL5	1.1 (0.93-1.4)	0.21	1.00000
HGF	1.7 (1.2-2.4)	0.0046	0.33120
GZMA	1.1 (0.71-1.8)	0.63	1.00000
HO1	1.1 (0.79-1.6)	0.51	1.00000
CX3CL1	0.92 (0.48-1.8)	0.8	1.00000
CXCL10	0.95 (0.78-1.2)	0.64	1.00000
CD70	1.2 (0.75-2.1)	0.4	1.00000
IL10	1.3 (1-1.7)	0.047	1.00000
TNFRSF12A	1.4 (0.92-2.2)	0.11	1.00000
CCL23	1.8 (1.1-2.9)	0.011	0.78100
CD5	0.91 (0.54-1.6)	0.74	1.00000
CCL3	1.3 (0.92-1.8)	0.14	1.00000
MMP7	2 (1.1-3.5)	0.016	1.00000
NCR1	1.1 (0.72-1.7)	0.65	1.00000
DCN	1.1 (0.49-2.3)	0.89	1.00000
TNFRSF21	1.1 (0.52-2.5)	0.74	1.00000
TNFRSF4	1 (0.66-1.6)	0.88	1.00000
MICAB	1 (0.88-1.2)	0.77	1.00000
CCL17	0.93 (0.75-1.1)	0.46	1.00000
ANGPT2	1.5 (0.88-2.4)	0.14	1.00000
PTN	0.91 (0.78-1.1)	0.25	1.00000
LAMP3	0.91 (0.66-1.3)	0.55	1.00000
CASP8	1.5 (1-2.1)	0.035	1.00000
ICOSLG	0.56 (0.23-1.4)	0.21	1.00000
MMP12	1.1 (0.86-1.4)	0.52	1.00000

CXCL13	1 (0.76-1.4)	0.87	1.00000
PDL2	1.3 (0.78-2.3)	0.3	1.00000
VEGFA	1.7 (1-2.8)	0.039	1.00000
IL12RB1	1.3 (0.83-2.1)	0.24	1.00000
CCL20	1.1 (0.99-1.3)	0.083	1.00000
KLRD1	0.87 (0.59-1.3)	0.48	1.00000
GZMB	0.96 (0.75-1.2)	0.74	1.00000
CD83	1.1 (0.64-1.8)	0.78	1.00000
IL12	1.3 (0.93-1.8)	0.12	1.00000
CSF1	6.7 (2.5-18)	0.00017	0.01326

CI: confidence interval; HR: hazard ratio

$ Bonferroni-Holm method

Supplementary Table 6: Association to survival, univariable Cox regression analysis

1.2.7 Supplementary Table 7

Variable	HR (95% CI)	p-value	HR (95% CI)	p-value	HR (95% CI)	p-value
TRAIL	0.30 (0.14-0.63)	0.001*	0.23 (0.10-0.55)	<0.001*	0.22 (0.10-0.53)	<0.001*
CSF1	5.88 (1.51-22.88)	0.011*	8.96 (1.67-48.17)	0.011*	10.61 (2.37-47.47)	0.002*
TIE2	3.69 (1.39-9.80)	0.009*	1.32 (0.36-4.84)	0.672		
T≥3	1.15 (0.62-2.13)	0.653	1.39 (0.68-2.82)	0.367	1.47 (0.76-2.82)	0.249
N1	2.28 (1.22-4.27)	0.010*	1.83 (0.91-3.68)	0.092	1.77 (0.89-3.51)	0.103
LV1	0.52 (0.20-1.34)	0.176	0.44 (0.15-1.24)	0.118	0.44 (0.15-1.25)	0.123
Pn1	0.54 (0.18-1.59)	0.263	0.98 (0.24-4.10)	0.981	1.01 (0.24-4.18)	0.989
R1	1.20 (0.52-2.76)	0.668	1.27 (0.49-3.24)	0.623	1.34 (0.54-3.31)	0.528
Grade≥2	3.84 (1.44-10.27)	0.007*	1.92 (0.60-6.17)	0.274	1.78 (0.58-5.47)	0.311
ASA≥3	1.92 (0.99-3.69)	0.052	2.20 (1.01-4.80)	0.059	2.18 (1.00-4.77)	0.051
Age (years)	1.00 (0.98-1.02)	0.876	0.99 (0.97-1.02)	0.489	0.99 (0.97-1.02)	0.508
Gender (female)	1.13 (0.62-2.06)	0.696	0.80 (0.36-1.77)	0.579	0.75 (0.36-1.58)	0.451
GPS≥1			2.28 (0.97-5.36)	0.059	2.36 (1.02-5.46)	0.044*

T≥3: Tumor extension stage 3-4; N1: lymph node metastasis; LV1: lymphovascular invasion; Pn1: perineural invasion; R1: microscopically tumor positive resection margin; Grade≥2: moderate or low tumor differentiation; ASA≥3: American Society of Anesthesiologists physical status class 3-4; GPS≥1: Glasgow prognostic score 1-2. * p<0.05

Supplementary Table 7: Multivariable Cox regression analyses including postoperative prognostic factors

1.2.8 Supplementary Table 8

Variable	iCCA (n=27) HR (95% CI)	p-value	pCCA (n=46) HR (95% CI)	p-value	GBC (n=29) HR (95% CI)	p-value
TRAIL	**0.13 (0.03-0.53)**	0.005*	**0.37 (0.14-0.97)**	0.044*	0.34 (0.08-1.47)	0.15
CSF1	**11.98 (1.43-100.3)**	0.022*	3.14 (0.63-15.73)	0.17	**20.25 (2.60-158.8)**	0.04*
TIE2	1.56 (0.40-6.21)	0.52	3.51 (0.99-12.50)	0.05	**18.96 (3.96-90.88)**	<0.001*

BTC: biliary tract cancer; CSF1: colony-stimulating factor 1; CI: confidence interval; GBC: gallbladder cancer; HR: hazard ratio; iCCA: intrahepatic cholangiocarcinoma; pCCA: perihilar cholangiocarcinoma; TIE2: tyrosine kinase with immunoglobulin-like and EGF-like domains 2; TRAIL: TNF-related apoptosis-inducing ligand

* p<0.05

Supplementary Table 8: Univariable Cox regression analyses for BTC subgroups

1.2.9 Supplementary Table 9

Protein	BTC (mean NPX)	Benign (mean NPX)	p-value (adjusted)$
IL6	4.31785201550388	3.5177034375	0.00022
PGF	8.00691697674419	7.73043	0.00029
CSF-1	7.99314286821705	7.8198128125	0.00081
MMP12	6.01492930232558	5.1874209375	0.0014
HGF	8.22759147286822	7.78830875	0.0017
CAIX	4.87341860465116	4.2741171875	0.0020
CCL20	6.38958573643411	5.1411440625	0.0020
CD70	3.09978302325581	2.8272303125	0.0026
CD4	1.10386023255814	0.8718778125	0.0046
MMP7	9.64063713178295	9.3349165625	0.0047
NCR1	3.05443496124031	2.74442	0.0049
CCL23	10.5575175193798	10.1484090625	0.0049
TNFRSF9	5.9465603875969	5.633436875	0.0077
ANGPT2	5.4263376744186	5.1509403125	0.0093
TNFRSF21	7.86686658914729	7.6957075	0.0096
TIE2	7.58498023255814	7.4245678125	0.011
TNFRSF12A	6.20066620155039	5.8256775	0.017
VEGFA	8.49613790697674	8.2632553125	0.019
IL12	6.87791511627907	6.3817490625	0.022
TNFRSF4	3.67786744186047	3.406905	0.022
LAP TGF-beta-1	1.77426534883721	1.5513334375	0.030
PD-L1	4.80189062015504	4.58476625	0.045
GZMA	5.60500782945736	5.32448	0.045
CD40	11.558720620155	11.231069375	0.045
IL2	-0.08507	-0.282786666666667	0.045
CXCL9	8.20772651162791	7.766839375	0.065
CX3CL1	5.73219527131783	5.57621625	0.065
TNFSF14	4.59257348837209	4.25287125	0.065
ADA	2.99782434108527	2.777410625	0.065
IL8	7.23265720930233	6.5674575	0.101
IL12RB1	1.88156294573643	1.6866115625	0.101
TWEAK	8.11264744186047	8.2794478125	0.155
CXCL13	8.81440503875969	8.4969921875	0.158
CD8A	9.70861612403101	9.3964153125	0.165
Gal-9	7.81087635658915	7.68446125	0.165
MCP-1	10.6923346511628	10.48241125	0.166
PD-L2	2.19872976744186	2.0584125	0.192
CCL19	10.1989911627907	9.9441334375	0.192
IL4	0.198068461538462	0.97131125	0.251
CXCL11	6.53334100775194	6.111983125	0.253
CD83	2.63795643410853	2.5222546875	0.273
KLRD1	6.16575410852713	5.993060625	0.285
PTN	4.3499992248062	3.8560209375	0.299
CD27	7.74662519379845	7.62677375	0.310
IL18	9.01281139534884	8.8128490625	0.310
Gal-1	6.33894674418605	6.284949375	0.329
CD5	4.70967782945736	4.6146853125	0.375
CD244	5.93353565891473	5.822475625	0.393
VEGFR-2	6.67078860465116	6.7173240625	0.393
MCP-4	6.52308426356589	6.682968125	0.393
FASLG	5.49450705426357	5.3801390625	0.393
TRAIL	7.71252937984496	7.7997078125	0.393
CD28	1.88470709090909	1.80059785714286	0.393
TNF	2.32800736842105	1.381855	0.451
CCL3	6.13909054263566	5.9741134375	0.457
VEGFC	0.648148914728682	0.8142946875	0.492
CXCL1	9.60512434108527	9.447909375	0.492
MIC-A/B	3.97844658914729	3.698789375	0.518
IL10	3.47482968992248	3.323515	0.567
CRTAM	4.60811596899225	4.5095509375	0.567
NOS3	1.17296548387097	1.0517128125	0.567
MCP-3	2.04767689922481	1.9040846875	0.567
CXCL10	7.95885751937984	7.767555625	0.608
ANG-1	8.27908100775194	8.4308478125	0.609
HO-1	12.1570479069767	12.0593178125	0.615
GZMB	3.43654573643411	3.662099375	0.618
ICOSLG	4.9059084496124	4.9383990625	0.638
CCL17	7.25083418604651	7.409985	0.649

CASP-8	4.23160472868217	4.153155	0.669
DCN	4.58040364341085	4.5493125	0.689
GZMH	5.25402054263566	5.138384375	0.693
IL7	5.03467356589147	4.9583621875	0.693
CXCL5	11.3869652713178	11.26014125	0.693
EGF	8.01752387596899	8.14532375	0.709
IL5	2.28320217948718	2.08642947368421	0.709
ARG1	3.75538125	3.89837	0.722
MCP-2	7.0301407751938	6.964016875	0.730
CXCL12	1.50443321428571	1.44411125	0.820
CCL4	7.55066410852713	7.5908634375	0.881
PDGF subunit B	10.0492405426357	10.0901725	0.892
PDCD1	2.88856046511628	2.8618746875	0.894
LAMP3	4.52632015503876	4.4987709375	0.900
IFN-beta	1.19878428571429	1.24540333333333	0.924
CD40-L	6.28631108527132	6.315771875	0.924
ADGRG1	1.75818330708661	1.77952892857143	0.935
FGF2	1.28708298387097	1.28857870967742	0.993

BTC: biliary tract cancer; NPX: normalized protein expression

$ Benjamini-Hochberg method

Supplementary Table 9: Differential expression between patients with BTC and patients with benign lesions

1.2.10 Supplementary Table 10

	Malignant	Healthy cholangiocyte	Hepatocyte	Fibroblast	Endothelial cell	CD4 T	CD8 T	Other T	NK	B	Macrophage	DC
**Zhang et al.**												
CSF1	0.0847	0.0829	0.0511	0.2819	0.4760	0.0636	0.0738	0.1024	0.1214	0.0096	0.0426	0.0108
TNFSF10	1.8147	1.2571	0.9562	0.2471	2.7682	0.9119	0.5068	0.6467	0.5525	0.3212	1.6501	0.5807
TEK	0.0023	0.0018	0.0174	0.0347	0.8827	0.0032	0.0050	0.0028	0.0009	0.0046	0.0029	0.0024
CSF1R	0.0069	0.0040	0.0058	0.0208	0.0574	0.0286	0.0247	0.0271	0.0422	0.0767	1.6110	0.5663
TNFRSF10A	0.0758	0.0484	0.0133	0.0132	0.1246	0.0466	0.0266	0.0405	0.0230	0.0440	0.0429	0.0299
TNFRSF10B	0.2084	0.1437	0.0723	0.1753	0.5363	0.0986	0.0888	0.1261	0.1175	0.0757	0.1750	0.1784
TNFRSF10C	0.0113	0.0542	0.0030	0.0035	0.0620	0.0010	0.0009	0.0034	0.0027	0.0016	0.0137	0.0048
TNFRSF10D	0.0411	0.0202	0.0142	0.0919	0.4605	0.0067	0.0049	0.0046	0.0078	0.0101	0.0345	0.0077
ANGPT1	0.0146	0.0014	0.0014	0.4345	0.0032	0.0021	0.0006	0.0009	0.0000	0.0000	0.0028	0.0020
ANGPT2	0.0099	0.0188	0.0175	1.4075	0.7765	0.0541	0.0632	0.0441	0.0781	0.0635	0.0090	0.0140
**Song et al.**												
CSF1	0.1506	0.0330		0.4926	0.2565	0.3552	0.4451	0.2652	0.2664	0.0352	0.1189	0.0295
TNFSF10	1.4861	2.0819		0.6649	2.2287	0.9558	0.5925	0.5782	0.9316	0.2805	2.2883	0.6189
TEK	0.0149	0.0217		0.0499	1.2572	0.0000	0.0013	0.0000	0.0000	0.0000	0.0054	0.0006
CSF1R	0.1134	0.0514		0.0851	0.0426	0.0096	0.0115	0.0028	0.0061	0.0178	1.6648	1.1076
TNFRSF10A	0.0887	0.1667		0.0241	0.1952	0.1037	0.0659	0.0538	0.0268	0.0740	0.0575	0.0226
TNFRSF10B	0.4983	0.7556		0.3614	0.8917	0.1977	0.1809	0.1820	0.1190	0.1711	0.2714	0.3389
TNFRSF10C	0.0524	0.0028		0.0057	0.0809	0.0004	0.0025	0.0063	0.0017	0.0017	0.7437	0.0085
TNFRSF10D	0.1330	0.1431		0.0457	0.5000	0.0398	0.0251	0.0223	0.0074	0.0197	0.0875	0.0287
ANGPT1	0.0006	0.0000		0.5088	0.0025	0.0004	0.0000	0.0011	0.0018	0.0000	0.0401	0.0106
ANGPT2	0.0482	0.0207		1.1587	1.0348	0.0364	0.0498	0.0294	0.0592	0.0634	0.0164	0.0258
ANGPT4	0.0033	0.0000		0.0157	0.0000	0.0000	0.0002	0.0000	0.0000	0.0000	0.0000	0.0000

B: B cell, CD4 T: cluster of differentiation 4 positive T cell; CD8 T: cluster of differentiation 8 positive T cell; DC: dendritic cell; NK: natural killer cell.

Supplementary Table 10: Cell type-specific expression of markers and receptors/ligands (average expression levels)

1.2.11 Supplementary Table 11

**malignant**					
	p_val	avg_log2FC	pct.1	pct.2	p_val_adj
CSF1	**5.16E-70**	**-0.0034**	**0.102**	**0.042**	**1.02E-65**
TNFSF10	**0.00E+00**	**0.5174**	**0.639**	**0.277**	**0.00E+00#**
TEK	**1.06E-19**	**-0.0589**	**0.002**	**0.018**	**2.10E-15**
CSF1R	**1.46E-155**	**-0.3889**	**0.009**	**0.117**	**2.90E-151**
TNFRSF10A	**1.51E-198**	**0.0481**	**0.113**	**0.024**	**2.98E-194**
TNFRSF10B	**1.23E-303**	**0.0826**	**0.261**	**0.077**	**2.43E-299**
TNFRSF10C	2.89E-02	-0.0017	0.013	0.010	1.00E+00
TNFRSF10D	**4.71E-48**	**0.0086**	**0.055**	**0.020**	**9.34E-44**
ANGPT1	**2.27E-27**	**0.0065**	**0.022**	**0.006**	**4.49E-23**
ANGPT2	**5.24E-19**	**-0.1264**	**0.012**	**0.032**	**1.04E-14**
**healthy cholangiocyte**					
	p_val	avg_log2FC	pct.1	pct.2	p_val_adj
CSF1	**2.04E-13**	**-0.0055**	**0.089**	**0.053**	**4.04E-09**
TNFSF10	7.01E-06	0.0697	0.397	0.356	1.39E-01
TEK	**1.05E-08**	**-0.0508**	**0.001**	**0.015**	**2.07E-04**
CSF1R	**6.86E-57**	**-0.3399**	**0.004**	**0.101**	**1.36E-52**
TNFRSF10A	5.97E-02	-0.0003	0.051	0.043	1.00E+00
TNFRSF10B	2.49E-01	-0.0176	0.127	0.118	1.00E+00
TNFRSF10C	**6.21E-96**	**0.0645**	**0.052**	**0.007**	**1.23E-91**
TNFRSF10D	2.15E-03	-0.0248	0.019	0.029	1.00E+00
ANGPT1	1.52E-05	-0.0153	0.002	0.011	3.02E-01
ANGPT2	4.60E-04	-0.0939	0.017	0.029	1.00E+00
**hepatocyte**					
	p_val	avg_log2FC	pct.1	pct.2	p_val_adj
CSF1	7.73E-02	-0.0490	0.040	0.056	1.00E+00
TNFSF10	3.50E-05	-0.1460	0.257	0.361	6.94E-01
TEK	1.09E-01	-0.0247	0.007	0.014	1.00E+00
CSF1R	**2.05E-14**	**-0.3166**	**0.003**	**0.094**	**4.07E-10**
TNFRSF10A	1.44E-05	-0.0505	0.008	0.045	2.85E-01
TNFRSF10B	**7.21E-10**	**-0.1113**	**0.038**	**0.121**	**1.43E-05**
TNFRSF10C	3.16E-01	-0.0134	0.007	0.011	1.00E+00
TNFRSF10D	5.65E-03	-0.0319	0.010	0.029	1.00E+00
ANGPT1	3.95E-02	-0.0143	0.002	0.010	1.00E+00
ANGPT2	4.72E-02	-0.0897	0.015	0.028	1.00E+00
**fibroblast**					
	p_val	avg_log2FC	pct.1	pct.2	p_val_adj
CSF1	**2.08E-39**	**0.2424**	**0.198**	**0.054**	**4.12E-35**
TNFSF10	**1.36E-21**	**-0.8019**	**0.157**	**0.362**	**2.69E-17**
TEK	1.66E-03	0.0003	0.032	0.014	1.00E+00
CSF1R	**2.88E-07**	**-0.2933**	**0.023**	**0.093**	**5.71E-03**
TNFRSF10A	9.68E-04	-0.0503	0.012	0.044	1.00E+00
TNFRSF10B	8.59E-03	0.0236	0.161	0.118	1.00E+00
TNFRSF10C	8.75E-02	-0.0126	0.002	0.011	1.00E+00
TNFRSF10D	**1.61E-09**	**0.0765**	**0.076**	**0.028**	**3.19E-05**
ANGPT1	**0.00E+00**	**0.5140**	**0.256**	**0.006**	**0.00E+00#**
ANGPT2	**0.00E+00**	**1.1826**	**0.415**	**0.022**	**0.00E+00#**
**endothelial**					
	p_val	avg_log2FC	pct.1	pct.2	p_val_adj
CSF1	**8.52E-74**	**0.4606**	**0.181**	**0.051**	**1.69E-69**
TNFSF10	**8.97E-105**	**0.8423**	**0.630**	**0.349**	**1.78E-100**
TEK	**0.00E+00**	**0.9072**	**0.337**	**0.003**	**0.00E+00#**
CSF1R	**4.32E-12**	**-0.2463**	**0.030**	**0.095**	**8.56E-08**
TNFRSF10A	1.35E-04	0.1047	0.067	0.043	1.00E+00
TNFRSF10B	**1.40E-36**	**0.4270**	**0.233**	**0.115**	**2.77E-32**
TNFRSF10C	**9.20E-14**	**0.0720**	**0.034**	**0.010**	**1.82E-09**
TNFRSF10D	**0.00E+00**	**0.5165**	**0.221**	**0.021**	**0.00E+00#**
ANGPT1	2.82E-02	-0.0118	0.003	0.010	1.00E+00
ANGPT2	**3.62E-226**	**0.7502**	**0.191**	**0.022**	**7.18E-222**

avg_log2FC: average log2 fold change of expression; pct.1: proportion of cells expressing marker in cell type; pct.2: proportion of cells expressing marker in other cell types; p_val: p-value unadjusted; p_val_adj: p-value adjusted (Benjamini-Hochberg method). **Bold type** indicates adjusted p-value <0.05; # indicates statistically significant p-value rounded to 0 (<2.226×10^{-308})

Supplementary Table 11: Cell type-specific expression of markers and receptors/ligands (malignant cells, tumor stroma and surrounding liver tissue) Zhang et al.

1.2.12 Supplementary Table 12

**malignant**					
	p_val	avg_log2FC	pct.1	pct.2	p_val_adj
CSF1	6.58E-01	-0.1976	0.073	0.072	1.00E+00
TNFSF10	**6.01E-70**	**0.3415**	**0.412**	**0.247**	**2.20E-65**
TEK	3.09E-02	-0.0191	0.006	0.010	1.00E+00
CSF1R	**1.44E-14**	**-0.1686**	**0.035**	**0.072**	**5.26E-10**
TNFRSF10A	**3.63E-36**	**0.0294**	**0.071**	**0.028**	**1.33E-31**
TNFRSF10B	**3.57E-191**	**0.3084**	**0.263**	**0.086**	**1.31E-186**
TNFRSF10C	**3.69E-14**	**-0.0617**	**0.032**	**0.014**	**1.35E-09**
TNFRSF10D	**4.18E-129**	**0.1183**	**0.091**	**0.018**	**1.53E-124**
ANGPT1	4.73E-02	-0.0125	0.001	0.003	1.00E+00
ANGPT2	1.87E-01	-0.0372	0.018	0.022	1.00E+00
ANGPT4	**2.90E-07**	**0.0045**	**0.001**	**0.000**	**1.06E-02**
**healthy cholangiocyte**					
	p_val	avg_log2FC	pct.1	pct.2	p_val_adj
CSF1	2.84E-03	-0.3394	0.019	0.073	1.00E+00
TNFSF10	**1.54E-09**	**0.6238**	**0.442**	**0.260**	**5.63E-05**
TEK	9.84E-01	-0.0078	0.010	0.010	1.00E+00
CSF1R	8.40E-04	-0.2391	0.010	0.069	1.00E+00
TNFRSF10A	**1.28E-14**	**0.1275**	**0.126**	**0.031**	**4.70E-10**
TNFRSF10B	**3.15E-28**	**0.5119**	**0.335**	**0.099**	**1.15E-23**
TNFRSF10C	2.08E-01	-0.1270	0.005	0.016	1.00E+00
TNFRSF10D	5.37E-05	0.1215	0.068	0.024	1.00E+00
ANGPT1	4.39E-01	-0.0123	0.000	0.003	1.00E+00
ANGPT2	8.08E-01	-0.0729	0.019	0.022	1.00E+00
ANGPT4	8.51E-01	-0.0006	0.000	0.000	1.00E+00
**hepatocyte**					
	p_val	avg_log2FC	pct.1	pct.2	p_val_adj
**fibroblast**					
	p_val	avg_log2FC	pct.1	pct.2	p_val_adj
CSF1	**5.56E-17**	**0.1945**	**0.240**	**0.072**	**2.03E-12**
TNFSF10	4.26E-02	-0.2704	0.202	0.261	1.00E+00
TEK	3.80E-06	0.0316	0.044	0.010	1.39E-01
CSF1R	1.61E-01	-0.1932	0.044	0.069	1.00E+00
TNFRSF10A	4.48E-02	-0.0617	0.005	0.032	1.00E+00
TNFRSF10B	3.69E-05	0.1422	0.197	0.100	1.00E+00
TNFRSF10C	2.64E-01	-0.1227	0.005	0.016	1.00E+00
TNFRSF10D	4.84E-01	-0.0078	0.033	0.025	1.00E+00
ANGPT1	**0.00E+00**	**0.5849**	**0.224**	**0.002**	**0.00E+00#**
ANGPT2	**6.07E-128**	**1.0159**	**0.279**	**0.020**	**2.22E-123**
ANGPT4	**4.16E-08**	**0.0221**	**0.005**	**0.000**	**1.52E-03**
**endothelial**					
	p_val	avg_log2FC	pct.1	pct.2	p_val_adj
CSF1	1.35E-05	-0.0560	0.120	0.072	4.96E-01
TNFSF10	**1.17E-60**	**0.7034**	**0.530**	**0.256**	**4.28E-56**
TEK	**0.00E+00**	**1.1693**	**0.433**	**0.002**	**0.00E+00#**
CSF1R	**3.91E-08**	**-0.2542**	**0.015**	**0.070**	**1.43E-03**
TNFRSF10A	**1.36E-31**	**0.1648**	**0.111**	**0.030**	**4.97E-27**
TNFRSF10B	**3.50E-128**	**0.6308**	**0.379**	**0.095**	**1.28E-123**
TNFRSF10C	8.51E-04	-0.0183	0.032	0.015	1.00E+00
TNFRSF10D	**1.38E-207**	**0.5247**	**0.208**	**0.021**	**5.04E-203**
ANGPT1	5.08E-01	-0.0088	0.002	0.003	1.00E+00
ANGPT2	**2.06E-182**	**0.9480**	**0.182**	**0.018**	**7.55E-178**
ANGPT4	7.34E-01	-0.0006	0.000	0.000	1.00E+00

avg_log2FC: average log2 fold change of expression; pct.1: proportion of cells expressing marker in cell type; pct.2: proportion of cells expressing marker in other cell types; p_val: p-value unadjusted; p_val_adj: p-value adjusted (Benjamini-Hochberg method). **Bold type** indicates adjusted p-value <0.05; # indicates statistically significant adjusted p-value rounded to 0 ($<2.226\times10^{-308}$)

Supplementary Table 12: Cell type-specific expression of markers and receptors/ligands (malignant cells, tumor stroma and surrounding liver tissue) Song et al.

1.2.13 Supplementary Table 13

**CD4 T: tumor vs surrounding liver**					
	p_val	avg_log2FC	pct.1	pct.2	p_val_adj
CSF1	**1.04E-21**	**0.1211**	**0.082**	**0.014**	**2.06E-17**
TNFSF10	**1.18E-39**	**0.9421**	**0.425**	**0.163**	**2.33E-35**
TEK	5.42E-04	0.0107	0.008	0.001	1.00E+00
CSF1R	2.08E-02	-0.0007	0.019	0.008	1.00E+00
TNFRSF10A	**8.34E-09**	**0.0163**	**0.050**	**0.013**	**1.65E-04**
TNFRSF10B	**1.36E-38**	**0.1430**	**0.144**	**0.023**	**2.69E-34**
TNFRSF10C	**2.46E-07**	**0.0107**	**0.008**	**0.000**	**4.88E-03**
TNFRSF10D	**4.24E-12**	**0.0275**	**0.021**	**0.001**	**8.39E-08**
ANGPT1	7.30E-03	0.0045	0.006	0.001	1.00E+00
ANGPT2	1.88E-01	-0.0157	0.023	0.015	1.00E+00
**CD8 T: tumor vs surrounding liver**					
	p_val	avg_log2FC	pct.1	pct.2	p_val_adj
CSF1	**5.00E-70**	**0.4475**	**0.156**	**0.012**	**9.90E-66**
TNFSF10	**1.21E-40**	**0.5383**	**0.429**	**0.142**	**2.39E-36**
TEK	4.34E-01	-0.0078	0.000	0.002	1.00E+00
CSF1R	9.53E-02	-0.0091	0.017	0.008	1.00E+00
TNFRSF10A	**8.61E-07**	**0.0349**	**0.037**	**0.009**	**1.71E-02**
TNFRSF10B	**3.47E-10**	**0.0718**	**0.091**	**0.028**	**6.87E-06**
TNFRSF10C	**4.79E-13**	**0.0174**	**0.011**	**0.000**	**9.50E-09**
TNFRSF10D	**7.34E-07**	**0.0365**	**0.014**	**0.001**	**1.45E-02**
ANGPT1	7.83E-01	-0.0009	0.000	0.000	1.00E+00
ANGPT2	4.18E-01	-0.0645	0.014	0.020	1.00E+00
**Other T: tumor vs surrounding liver**					
	p_val	avg_log2FC	pct.1	pct.2	p_val_adj
CSF1	**3.89E-07**	**0.0460**	**0.099**	**0.021**	**7.70E-03**
TNFSF10	**2.37E-75**	**1.4574**	**0.652**	**0.071**	**4.69E-71**
TEK	6.46E-01	-0.0049	0.000	0.001	1.00E+00
CSF1R	1.90E-01	-0.0467	0.000	0.009	1.00E+00
TNFRSF10A	2.95E-03	0.0199	0.044	0.012	1.00E+00
TNFRSF10B	**4.51E-08**	**0.1466**	**0.133**	**0.032**	**8.93E-04**
TNFRSF10C	1.45E-05	0.0277	0.022	0.000	2.87E-01
TNFRSF10D	2.56E-02	0.0163	0.011	0.001	1.00E+00
ANGPT1	3.05E-02	0.0077	0.006	0.000	1.00E+00
ANGPT2	6.52E-01	-0.0541	0.011	0.015	1.00E+00
**NK: tumor vs surrounding liver**					
	p_val	avg_log2FC	pct.1	pct.2	p_val_adj
CSF1	**3.45E-13**	**0.4020**	**0.157**	**0.025**	**6.84E-09**
TNFSF10	**3.90E-06**	**0.4174**	**0.330**	**0.148**	**7.73E-02**
TEK	7.71E-01	-0.0015	0.000	0.001	1.00E+00
CSF1R	2.51E-01	0.0016	0.026	0.013	1.00E+00
TNFRSF10A	**1.37E-06**	**0.0824**	**0.052**	**0.006**	**2.71E-02**
TNFRSF10B	9.50E-02	-0.0027	0.070	0.036	1.00E+00
TNFRSF10C	7.71E-01	-0.0042	0.000	0.001	1.00E+00
TNFRSF10D	7.96E-03	0.0374	0.017	0.002	1.00E+00
ANGPT1	1.00E+00	0.0000	0.000	0.000	1.00E+00
ANGPT2	1.52E-02	0.0136	0.061	0.022	1.00E+00
**B: tumor vs surrounding liver**					
	p_val	avg_log2FC	pct.1	pct.2	p_val_adj
CSF1	1.24E-02	0.0214	0.021	0.004	1.00E+00
TNFSF10	**1.08E-06**	**0.2201**	**0.271**	**0.117**	**2.13E-02**
TEK	4.03E-02	0.0085	0.011	0.001	1.00E+00
CSF1R	3.69E-05	0.0463	0.101	0.030	7.32E-01
TNFRSF10A	7.34E-01	-0.0417	0.016	0.019	1.00E+00
TNFRSF10B	**1.39E-06**	**0.0972**	**0.106**	**0.026**	**2.76E-02**
TNFRSF10C	4.19E-03	0.0119	0.011	0.000	1.00E+00
TNFRSF10D	5.33E-04	0.0431	0.027	0.003	1.00E+00
ANGPT1	1.00E+00	0.0000	0.000	0.000	1.00E+00
ANGPT2	3.39E-01	-0.0252	0.048	0.032	1.00E+00
**Dendritic: tumor vs surrounding liver**					
	p_val	avg_log2FC	pct.1	pct.2	p_val_adj
CSF1	2.76E-05	0.0346	0.027	0.003	5.46E-01
TNFSF10	9.27E-01	-0.0991	0.294	0.268	1.00E+00
TEK	2.15E-01	0.0051	0.006	0.002	1.00E+00
CSF1R	**1.31E-17**	**0.3935**	**0.492**	**0.224**	**2.59E-13**

	p_val	avg_log2FC	pct.1	pct.2	p_val_adj
TNFRSF10A	3.21E-01	0.0086	0.030	0.020	1.00E+00
TNFRSF10B	**4.09E-16**	**0.2281**	**0.267**	**0.087**	**8.10E-12**
TNFRSF10C	2.16E-01	-0.0006	0.006	0.002	1.00E+00
TNFRSF10D	1.31E-04	0.0221	0.021	0.002	1.00E+00
ANGPT1	7.98E-02	0.0059	0.006	0.001	1.00E+00
**Macrophage: tumor vs surrounding liver**					
	p_val	avg_log2FC	pct.1	pct.2	p_val_adj
CSF1	4.97E-03	0.0189	0.038	0.022	1.00E+00
TNFSF10	**3.40E-20**	**-0.4580**	**0.421**	**0.514**	**6.73E-16**
TEK	3.51E-01	-0.0043	0.001	0.003	1.00E+00
CSF1R	2.37E-02	-0.1780	0.580	0.532	1.00E+00
TNFRSF10A	2.37E-04	-0.0006	0.043	0.021	1.00E+00
TNFRSF10B	**2.34E-12**	**0.0889**	**0.159**	**0.080**	**4.64E-08**
TNFRSF10C	1.20E-01	0.0099	0.012	0.007	1.00E+00
TNFRSF10D	3.93E-03	0.0138	0.035	0.019	1.00E+00
ANGPT1	5.17E-02	0.0051	0.004	0.001	1.00E+00
ANGPT2	2.02E-01	0.0042	0.008	0.005	1.00E+00

avg_log2FC: average log2 fold change of expression; pct.1: proportion of cells expressing marker in cell type, tumor tissue; pct.2: proportion of cells expressing marker in cell type, surrounding tissue; p_val: p-value unadjusted; p_val_adj: p-value adjusted (Benjamini-Hochberg method). **Bold type** indicates adjusted p-value <0.05

Supplementary Table 13: Immune cell type-specific expression of markers and receptors/ligands (tumor versus surrounding liver tissue) Zhang et al.

1.2.14 Supplementary Table 14

	p_val	avg_log2FC	pct.1	pct.2	p_val_adj
**CD4 T: tumor vs surrounding liver**					
	p_val	avg_log2FC	pct.1	pct.2	p_val_adj
CSF1	**2.34E-16**	**0.3807**	**0.110**	**0.051**	**8.55E-12**
TNFSF10	1.25E-04	0.2607	0.251	0.213	1.00E+00
TEK	1.00E+00	0.0000	0.000	0.000	1.00E+00
CSF1R	1.04E-01	0.0143	0.005	0.002	1.00E+00
TNFRSF10A	2.22E-03	0.0800	0.043	0.027	1.00E+00
TNFRSF10B	1.80E-01	-0.0066	0.059	0.068	1.00E+00
TNFRSF10C	2.99E-01	-0.0011	0.000	0.000	1.00E+00
TNFRSF10D	**2.77E-07**	**0.0696**	**0.020**	**0.004**	**1.02E-02**
ANGPT1	3.36E-01	0.0012	0.000	0.000	1.00E+00
ANGPT2	3.40E-04	-0.0356	0.008	0.019	1.00E+00
ANGPT4	1.00E+00	0.0000	0.000	0.000	1.00E+00
**CD8 T: tumor vs surrounding liver**					
	p_val	avg_log2FC	pct.1	pct.2	p_val_adj
CSF1	**2.96E-84**	**0.6626**	**0.156**	**0.051**	**1.09E-79**
TNFSF10	**4.58E-15**	**0.2440**	**0.197**	**0.144**	**1.68E-10**
TEK	6.63E-01	-0.0009	0.000	0.000	1.00E+00
CSF1R	1.02E-01	0.0077	0.005	0.003	1.00E+00
TNFRSF10A	2.19E-03	0.0565	0.026	0.018	1.00E+00
TNFRSF10B	2.09E-01	-0.0038	0.052	0.057	1.00E+00
TNFRSF10C	9.15E-01	-0.0007	0.000	0.001	1.00E+00
TNFRSF10D	**1.51E-12**	**0.0683**	**0.014**	**0.003**	**5.54E-08**
ANGPT1	1.00E+00	0.0000	0.000	0.000	1.00E+00
ANGPT2	1.79E-04	-0.0393	0.009	0.018	1.00E+00
ANGPT4	1.77E-01	0.0007	0.000	0.000	1.00E+00
**Other T: tumor vs surrounding liver**					
	p_val	avg_log2FC	pct.1	pct.2	p_val_adj
CSF1	1.93E-02	0.2453	0.104	0.076	1.00E+00
TNFSF10	5.96E-01	0.0648	0.193	0.210	1.00E+00
TEK	1.00E+00	0.0000	0.000	0.000	1.00E+00
CSF1R	8.47E-01	-0.0016	0.002	0.002	1.00E+00
TNFRSF10A	1.43E-05	0.0971	0.046	0.016	5.23E-01
TNFRSF10B	8.29E-01	0.0042	0.070	0.074	1.00E+00
TNFRSF10C	6.89E-01	0.0015	0.002	0.002	1.00E+00
TNFRSF10D	**2.38E-06**	**0.0868**	**0.024**	**0.004**	**8.71E-02**
ANGPT1	5.78E-01	-0.0021	0.000	0.000	1.00E+00
ANGPT2	7.50E-02	-0.0301	0.006	0.016	1.00E+00
ANGPT4	1.00E+00	0.0000	0.000	0.000	1.00E+00
**NK: tumor vs surrounding liver**					
	p_val	avg_log2FC	pct.1	pct.2	p_val_adj
CSF1	**3.51E-104**	**0.8810**	**0.175**	**0.011**	**1.28E-99**
TNFSF10	5.82E-05	-0.1109	0.225	0.289	1.00E+00
TEK	1.00E+00	0.0000	0.000	0.000	1.00E+00
CSF1R	1.43E-01	-0.0055	0.001	0.003	1.00E+00
TNFRSF10A	1.27E-05	0.0600	0.023	0.007	4.66E-01
TNFRSF10B	4.43E-01	0.0112	0.041	0.047	1.00E+00
TNFRSF10C	9.24E-01	0.0000	0.001	0.001	1.00E+00
TNFRSF10D	3.99E-06	0.0282	0.010	0.001	1.46E-01
ANGPT1	9.24E-01	-0.0005	0.001	0.001	1.00E+00
ANGPT2	3.87E-01	-0.0031	0.019	0.023	1.00E+00
ANGPT4	1.00E+00	0.0000	0.000	0.000	1.00E+00
**B: tumor vs surrounding liver**					
	p_val	avg_log2FC	pct.1	pct.2	p_val_adj
CSF1	5.37E-01	0.0212	0.021	0.016	1.00E+00
TNFSF10	9.09E-01	0.0003	0.133	0.130	1.00E+00
TEK	1.00E+00	0.0000	0.000	0.000	1.00E+00
CSF1R	2.34E-02	0.0203	0.021	0.007	1.00E+00
TNFRSF10A	6.89E-01	-0.0134	0.031	0.034	1.00E+00
TNFRSF10B	7.14E-01	0.0755	0.069	0.066	1.00E+00
TNFRSF10C	8.49E-02	-0.0049	0.000	0.004	1.00E+00
TNFRSF10D	5.75E-01	0.0195	0.011	0.008	1.00E+00
ANGPT1	1.00E+00	0.0000	0.000	0.000	1.00E+00
ANGPT2	3.07E-02	-0.0532	0.019	0.038	1.00E+00
ANGPT4	1.00E+00	0.0000	0.000	0.000	1.00E+00
**Dendritic: tumor vs surrounding liver**					

	p_val	avg_log2FC	pct.1	pct.2	p_val_adj
CSF1	5.19E-01	0.0540	0.030	0.023	1.00E+00
TNFSF10	7.43E-01	0.0258	0.403	0.433	1.00E+00
TEK	4.59E-01	-0.0013	0.000	0.002	1.00E+00
CSF1R	**9.64E-16**	**0.6869**	**0.604**	**0.353**	**3.53E-11**
TNFRSF10A	5.10E-01	-0.0045	0.026	0.035	1.00E+00
TNFRSF10B	2.18E-01	0.0526	0.265	0.322	1.00E+00
TNFRSF10C	4.04E-01	-0.0054	0.007	0.014	1.00E+00
TNFRSF10D	8.28E-01	-0.0207	0.030	0.027	1.00E+00
ANGPT1	8.94E-02	-0.0178	0.004	0.019	1.00E+00
ANGPT2	5.08E-01	-0.0018	0.022	0.031	1.00E+00
ANGPT4	1.00E+00	0.0000	0.000	0.000	1.00E+00
**Macrophage: tumor vs surrounding liver**					
	p_val	avg_log2FC	pct.1	pct.2	p_val_adj
CSF1	**2.50E-11**	**0.1949**	**0.052**	**0.014**	**9.16E-07**
TNFSF10	**2.72E-28**	**-0.4684**	**0.394**	**0.511**	**9.96E-24**
TEK	3.89E-02	-0.0138	0.001	0.004	1.00E+00
CSF1R	**1.05E-34**	**0.5555**	**0.527**	**0.361**	**3.86E-30**
TNFRSF10A	4.47E-02	0.0034	0.047	0.034	1.00E+00
TNFRSF10B	3.51E-01	0.0161	0.162	0.149	1.00E+00
TNFRSF10C	**1.63E-31**	**-0.7181**	**0.046**	**0.154**	**5.95E-27**
TNFRSF10D	**6.23E-09**	**0.1061**	**0.070**	**0.030**	**2.28E-04**
ANGPT1	3.71E-02	-0.0107	0.007	0.013	1.00E+00
ANGPT2	5.15E-01	0.0027	0.011	0.013	1.00E+00
ANGPT4	1.00E+00	0.0000	0.000	0.000	1.00E+00

avg_log2FC: average log2 fold change of expression; pct.1: proportion of cells expressing marker in cell type, tumor tissue; pct.2: proportion of cells expressing marker in cell type, surrounding tissue; p_val: p-value unadjusted; p_val_adj: p-value adjusted (Benjamini-Hochberg method). **Bold type** indicates adjusted p-value <0.05

Supplementary Table 14: Immune cell type-specific expression of markers and receptors/ligands (tumor versus surrounding liver tissue) Song et al.
